# SURF1 deficiency: a multi-centre natural history study

**DOI:** 10.1186/1750-1172-8-96

**Published:** 2013-07-05

**Authors:** Yehani Wedatilake, Ruth M Brown, Robert McFarland, Joy Yaplito-Lee, Andrew A M Morris, Mike Champion, Phillip E Jardine, Antonia Clarke, David R Thorburn, Robert W Taylor, John M Land, Katharine Forrest, Angus Dobbie, Louise Simmons, Erlend T Aasheim, David Ketteridge, Donncha Hanrahan, Anupam Chakrapani, Garry K Brown, Shamima Rahman

**Affiliations:** 1 Mitochondrial Research Group, UCL Institute of Child Health, London, UK; 2 Department of Biochemistry, University of Oxford, Oxford, UK; 3 Wellcome Trust Centre for Mitochondrial Research, Newcastle University, Newcastle-upon-Tyne, UK; 4 Murdoch Childrens Research Institute, Royal Children’s Hospital, Melbourne, Australia; 5 Central Manchester University Hospital, Manchester, UK; 6 Evelina Children’s Hospital, London, UK; 7 Bristol Royal Hospital for Children, Bristol, UK; 8 St George’s Hospital NHS Trust, London, UK; 9 Neurometabolic Unit, National Hospital for Neurology and Neurosurgery, London, UK; 10 Southampton University Hospitals NHS Foundation Trust, Southampton, UK; 11 Yorkshire Regional Genetics Service, Leeds, UK; 12 Birmingham Children’s Hospital, Birmingham, UK; 13 MRC Epidemiology Unit, Institute of Metabolic Science, Addenbrooke’s Hospital, Cambridge, UK; 14 Women’s & Children’s Hospital, Adelaide, Australia; 15 Royal Belfast Hospital for Sick Children, Belfast, Ireland; 16 Metabolic Unit, Great Ormond Street Hospital, London, UK

**Keywords:** SURF1, Leigh syndrome, Cytochrome *c* oxidase, Complex IV, Mitochondrial disease

## Abstract

**Background:**

SURF1 deficiency, a monogenic mitochondrial disorder, is the most frequent cause of cytochrome *c* oxidase (COX) deficient Leigh syndrome (LS). We report the first natural history study of SURF1 deficiency.

**Methods:**

We conducted a multi-centre case notes review of 44 SURF1-deficient patients from ten different UK centres and two Australian centres. Survival data for LRPPRC-deficient LS and nuclear-encoded complex I-deficient LS patients were obtained from previous publications. The survival of SURF1-deficient patients was compared with these two groups using Kaplan**-**Meier survival analysis and logrank test.

**Results:**

The majority of patients (32/44, 73%) presented in infancy (median 9.5 months). Frequent symptoms were poor weight gain (95%, median age 10 months), hypotonia (93%, median age 14 months), poor feeding/vomiting (89%, median age 10 months), developmental delay (88%, median age 14 months), developmental regression (71%, median age 19 months), movement disorder (52%, median age 24 months), oculomotor involvement (52%, median age 29 months) and central respiratory failure (78%, median age 31 months). Hypertrichosis (41%), optic atrophy (23%), encephalopathy (20%), seizures (14%) and cardiomyopathy (2%) were observed less frequently.

Lactate was elevated in CSF (mean 4.3 mmol/L) in all patients (30/30) and in blood (mean 4.4 mmol/L) in 31/38 (81%). Fibroblast COX activity was universally decreased (25/25). Normal COX histochemistry was noted in 30% of biopsies, whereas muscle COX activity was reduced in 96% (25/26). Neuroimaging demonstrated lesions characteristic of LS in 28/33 (85%) and atypical findings in 3/33 (9%). Peripheral neuropathy was present in 13/16 (81%) (demyelinating 7/16, axonal 2/16). Kaplan-Meier analysis demonstrated that SURF1-deficient patients experience longer survival (median 5.4 years, p < 0.001) compared to LRPPRC deficiency (median 1.8 years) and nuclear-encoded complex I-deficient LS (median 1.6 years). Survival >10 years was observed in 7 patients, 6 of these patients did not experience neurological regression. The most frequent mutation was c.312_320del10insAT. Five novel mutations (c.468_469delTC, c.799_800delCT, c.575G>A (p.Arg192Gln), c.751+5G>A and c.752-2A>G) were identified.

**Conclusions:**

SURF1-deficient patients have a homogeneous clinical and biochemical phenotype. Early recognition is essential to expedite diagnosis and enable prenatal diagnosis.

## Background

SURF1 deficiency is a recessively inherited mitochondrial disorder and is the most frequent cause of Leigh syndrome (LS) associated with cytochrome *c* oxidase (COX, complex IV) deficiency. COX is the fourth complex of the mitochondrial oxidative phosphorylation (OXPHOS) system where sequential electron transfer is coupled to proton pumping by complexes I-IV. The electrochemical gradient generated is ultimately used by complex V (ATP synthase) to synthesise ATP from ADP and inorganic phosphate. A number of assembly factors are required for the intricate biogenesis of the COX holoenzyme and studies in *Paracoccus denitrificans* and *Saccharomyces cerevisiae* have identified SURF1 as a key player in the early assembly of COX [1,2].

Due to their clinical and genetic heterogeneity, mitochondrial diseases are often a diagnostic challenge. LS, a fatal subacute necrotising encephalomyelopathy, is a genetically heterogeneous mitochondrial disorder which can be associated with any OXPHOS deficiency [3]. Since *SURF1* mutations were linked to COX deficiency and LS in 1998 [4,5] isolated case reports and mutation series have been reported [6,7]. However a collective description depicting the natural history with biochemical and radiological features of this disease has not yet been published. The disease features of SURF1 deficiency are not widely recognized by paediatricians and a clear understanding of the clinical continuum of the disease is important to improve diagnosis of this condition. Affected families often enquire about prognosis, but there is limited data in the literature to provide prognostic information. Well-documented natural history studies are also an invaluable aid to planning future clinical trials.

We sought to characterise the phenotypic and genotypic spectrum of SURF1 deficiency, to provide survival data in a large cohort, and highlight clinical predictors of longer survival. We also report 5 novel mutations and review all the cases published in the literature.

## Materials and methods

We conducted a multi-centre case notes review of all patients diagnosed since 1998, when *SURF1* mutations were first linked to LS. All SURF1-deficient patients were identified through the NHS Specialized Services-funded mitochondrial molecular diagnostic laboratory in Oxford which provides a national diagnostic service for *SURF1* in the UK, and through the major Australasian paediatric diagnostic centre (Murdoch Childrens Research Institute, Melbourne). All subjects with a confirmed genetic diagnosis of SURF1 deficiency, defined as two pathogenic variants in the *SURF1* gene, were considered eligible. We also included patients who died before the *SURF1* gene was identified, if subsequent analysis of stored samples revealed two pathogenic *SURF1* mutations. Where stored samples were unavailable, we included deceased patients if they had an affected sibling with a confirmed *SURF1* mutant genotype and a similar disease course.

Clinical data including the age-of-onset of symptoms, neuroimaging, nerve conduction studies, muscle histology and histochemistry, blood lactate, CSF lactate, and respiratory chain enzyme (RCE) activities in muscle and cultured skin fibroblasts, were collected using a structured questionnaire completed by the responsible clinician at each centre. The questionnaire (Additional file 1) was developed and tested in a pilot study conducted at Great Ormond Street Hospital, London, UK on ten patients with genetically confirmed SURF1 deficiency.

All genetic studies were performed with informed consent of parents/legal guardians of the patients. Ethical approval for the study was obtained from the National Research Ethics Committee London Bloomsbury, UK and the Royal Children’s Hospital Human Research Ethics Committee, Melbourne, Australia (HREC 32188A).

### Systematic literature review

To identify previously published cases a systematic literature search was conducted in PubMed in January 2013 using the search terms “SURF1”, “SURF-1”, “Leigh syndrome”, “cytochrome c oxidase deficiency”, “complex IV deficiency”, and “Cox deficiency”. The search was limited to studies in humans published after 1.1.1998 (the year SURF1 deficiency was first described). There were no language restrictions. Of 2135 identified records, 1969 were excluded based on review of titles, 110 were excluded on the basis of abstracts, and 56 full-text records were retrieved. Of these, 43 records describing 129 cases with SURF1 deficiency were reviewed. Bibliographies were searched manually for additional records. The cases were used to ascertain previously identified phenotypes and genotypes, to draw phenotype and genotype comparisons between our cohort and previously published cases and for Kaplan**-**Meier survival analysis. These cases were not included in our natural history analysis, as data was incomplete and the chronological onset of symptoms was not available in many of the published cases. Authors were contacted and raw data was obtained for survival rates of LRPPRC-deficient LS patients [8] and nuclear-encoded complex I-deficient LS patients [9].

### Statistical analysis

Kaplan**-**Meier survival analysis was used to compare survival rates for the SURF1 deficiency, LRPPRC-deficient LS and nuclear-encoded complex I-deficient LS patients. Mortality rates were compared using the log rank test. Statistical analysis was performed using Stata 12.1 software (Stata, College Station, Tex).

## Results

### Demography

We identified 57 patients with SURF1 disease born between 1976 and 2010, diagnosed at ten centres in the UK (n = 52) and two centres in Australia (n = 5). Genotypes were established in 54 patients and 3 deceased patients were assumed to have the same genotype as their affected sibling with SURF1 deficiency since they had a similar disease course. Detailed clinical data were available in 45 patients. It was not possible to trace clinical records and/or the responsible clinician in the remaining 12 cases. One patient from a consanguineous pedigree was found to have genetically confirmed Zellweger syndrome in addition to SURF1 disease (homozygous c.799_800delCT) and was excluded from further analyses. The study group consisted of 44 patients (24 males, 55%) from 37 pedigrees, with 10 (23%) patients from consanguineous families. Of these, 4 cases (cases 1b, 30, 35 and 36 in Table 1) have been published [3,10,11]. White Europeans formed the largest ethnic group (n = 20, 45%) followed by Bangladesh Bengali (n = 5, 11%), Indian Gujarati (n = 5, 11%) and Pakistani (n = 4, 9%). Other ethnicities included Turkish Cypriot (n = 2), Chinese (n = 1), Sri Lankan (n = 1) and six cases where the ethnicity was not specified.

**Table 1:** Clinical features and genotypes of 44 patients with SURF1 deficiency

**Case**	**Gender**	**Consanguinity**	**Clinical features**	Age at onset* (months)	Age at death* (months)	**Skeletal muscle histology/histochemistry**	**Muscle COX**	**Fibroblast COX activity**	**Abnormal regions on neuroimaging**	***SURF1 *Mutations**
1a	M	Yes	PF/V, PW, nystagmus, RF	8	2y 1 m	NA	NA	29% of LLR	Cp, tectal plate, PAG, Icp, Ion, Dt, corticospinal tracts	Homo c.792_793delAG
1b	F	Yes	PW, hypotonia, DR, DD, hypertrichosis, nystagmus, RF	9	3y	T1FP, increased lipid	Low	UD	Leukoencephalopathy: WM, posterior limbs of IC, Cc, Dt, cerebellar WM	Homo c.792_793delAG
2	F	No	Ataxia, hypotonia, nystagmus, ophthalmoplegia, PW, tremor	18	Alive 12y	Smaller T1 fibres	Low	19% of LLR	Pu, Me, Ico	c.240+1G >T, c.575G>A
3	F	No	DD, hypotonia, nystagmus, PW, tremor, ophthalmoplegia, ataxia	birth	Alive 19y	Normal	Low	37% of LLR	Mb, pons	c.312_320del10insAT, c.751+5G>A
4	F	No	PF, movement disorder, PW, DD, RF	7	16	NA	Low	57% of LLR	NA	Homo c.312_320del10insAT
5a	M	No	PF, ataxia, nystagmus. ophthalmoplegia, tremor, PW, DR, RF	10	5y 2 m	Absent COX, increased lipid	Low	UD	Dt, Cd, Mb, Me	Homo c.516-2A>G
5b	M	No	PF/V DD, DR, RF	3 days	20	Increased lipid	NA	NA	At 1y: normal	Homo c.516-2A>G
6	M	No	V, PW, hypotonia, DR, ophthalmoplegia, ataxia, encephalopathy	9	21	Increased lipid, reduced COX	NA	12% of LLR	Dt, dorsal BS, Cp, STh	Homo c.324-11T>G
7	M	No	V, PW, DR, tremor, nystagmus, hypotonia, ophthalmoplegia, ataxia	10	4y	Increased lipid	NA	27% of LLR	Th, Cd, GP BS, cerebellum	Homo c.312_320del10insAT
8a	F	No	Hypertrichosis, DD, ataxia, hypotonia, nystagmus, ophthalmoplegia, encephalopathy, PF, OA, RF	9	11y 9 m	Reduced COX, T1FP, increased lipid	NA	UD	CT brain: GP and cerebellar atrophy	Homo c.516-2A>G
8b	F	No	V, DD, PW, hypertrichosis, hypotonia, nystagmus, ophthalmoplegia, ataxia, RF	10	6y 6 m	NA	NA	NA	CT brain: normal	Homo c.516-2A>G
9a	M	No	V, PW, DD nystagmus, ophthalmoplegia, hypotonia	2	24 m	Increased lipid, reduced COX.	NA	27% of LLR	CT brain: normal	Homo c.312_320del10insAT
9b	F	No	Hypotonia, PF/V, PW, DD, hypertrichosis, motor delay, ophthalmoplegia, RF, OA	1.5	4y	NA	NA	NA	CT brain: widened subarachnoid spaces	Homo c.312_320del10insAT
10	M	Yes	Hypotonia, PF/V, DD, nystagmus, PW, ataxia, RF	4	24 m	T1FP	Low	50% of LLR	NA	Homo c.516-2A>G
11	F	Yes	DD, DR, PF/V, PW, tremor, OA, PW, hypotonia, nystagmus, ataxia, RF	9	7y 1 m	Absent COX increased lipid, T1FP	NA	7% of LLR	Mb, Dt, Cd, GP	Homo c.751C>T
12	M	No	PF/V, DD, DR, hypotonia, encephalopathy, PW, RF	6	13 m	NA	NA	NA	Cd, Pu, BS	Homo c.312_320del10insAT, c.688C>T
13a	F	Yes	DD, DR, PF/V, nystagmus, dystonia, PW, Sz, hypertrichosis, RF	12	5y 5 m	T1FP, Reduced COX	Low	53% of LLR	Me, Cc, cerebellar atrophy, multicystic changes in peritrigonal regions, posterior limbs of IC	Homo c.324-11 T>G
13b	F	Yes	PF/V, PW, hypotonia, DD, DR	15	Outcome unknown	NA	Low	NA	NA	Homo c.324-11 T>G
14	F	Yes	DD, DR, hypotonia, PF/V, chorea, PW, nystagmus	18	Outcome unknown	Reduced COX, increased lipid	Low	UD	BG, BS, long tracts	Homo c.792_793delAG
15	M	No	Hypotonia, DD, DR, dystonia, ophthalmoplegia, PF/V, RF	2y 6 m	4y 6 m	Reduced COX, T1FP	Low	UD	BS, Calcification of Dt, Cd, GP	Homo c.312_320del10insAT
16	M	Yes	PF/V, PW, hypotonia, DD, DR, encephalopathy, RF	9	18 m	Reduced COX.	Low	17% of LLR	BS, Dt, SN, Pu, GP, Cd, Cc, cystic changes in Dt, BG and central WM	Homo c.324-11T>G
17	M	No	Short stature, ataxia, PW, hypertrichosis, nystagmus, DD	5y	Alive 15y	Increased lipid, reduced COX	Low	NA	Dt and cerebellar atrophy	Homo c.312_320del10insAT
18	F	Yes	PF/V, DR, PW, DD, hypotonia, tremor, dystonia, OA, ophthalmoplegia	10	8y 9 m	Absent COX T1FP	Low	40% of LLR	Leukoencephalopathy: WM abnormalities in cerebral hemispheres and cerebellum	Homo c.833+1G>A
19	M	No	Hypotonia, PF/V, PW, choreoathetoid movements, dystonia, DR, RF	10	3y 6 m	Reduced COX T1FP	Low	NA	Cd, Cd, GP Cp, SN.	Homo c.312_320del10insAT
20	M	No	Hypotonia, apnoeic episodes, motor delay, ataxia, DR, DD, tremor, hypertrichosis, dystonia, PF/V, PW, RF ophthalmoplegia, nystagmus, hypertrichosis	10	Alive 16y	Reduced COX	Low	NA	At 1y: normal	Homo c.704C>T
21a	M	No	V, PW, generalized hypotonia, nystagmus, ophthalmoplegia, DD, encephalopathy, RF	10	2y 3 m	Absent COX, increased lipid	Low	23% of LLR	Pu, ventral Me, cervico-medullary region	Homo c.792_793delAG
21b	M	No	PF/V, PW, DD, DR, hypotonia, OA, ophthalmoplegia, nystagmus, hypertrichosis, RF	9	2y 5 m	NA	NA	NA	NA	Homo c.792_793delAG
22	M	No	DD, DR, PW, ataxia, dysarthria, choking episodes, V, hypotonia, nystagmus, hypertrophic cardiomyopathy	4y 3 m	Outcome unknown	Absent COX	Low	NA	Deep grey matter involvement of cerebellum, Mb, Pu	c.240+1G>T, c.574C>T
23	F	No	PF/V, DR, DD, PW, hypotonia, nystagmus, tremor, dystonia, hypertrichosis, ophthalmoplegia, encephalopathy, ataxia, RF	15	21 m	Absent COX	Low	NA	Cc, Mb, BS, GP	Homo c.312_320del10insAT
24	F	No	PW, V, hypertrichosis, DD, hypotonia, ophthalmoplegia, tremor, Sz, ataxia, encephalopathy, RF	2	2y 10 m	Absent COX	Low	NA	Thalamic fasciculus, RN, Mb, pons, Scp, Dt, Me, WM tracts of spinal cord	Homo c.324-11T>G
25a	M	No	PW, DD, ataxia, falls, OA, hypotonia, hypertrichosis, ophthalmoplegia, nystagmus, ataxia, RF	20	14y	NA	NA	NA	Me, Icp, pons. Linear area in the periventricular WM of both occipital lobes	Homo c.871insT
25b	M	No	DD, PF/V, PW, choreoathetosis, ataxia, hypotonia, OA, encephalopathy, ataxia, RF	24	11y	Normal	NA	30% of LLR	Cd, GP	Homo c.871insT
26	M	No	Hypotonia, V, PW, ophthalmoplegia, DD, nystagmus, dyskinesia, choreoathetoid movements, RF	2	3y 10 m	Increased lipid	NA	UD	Cd, Cd, GP, deep WM in cerebellar hemispheres	c.312_320del10insAT, c.240+1G>T
27	M	No	DD, hypotonia, PF, PW, DR, RF	10	4y 9 m	Reduced COX	Normal	UD	CT brain: BG	c.845_846delCT, c.240+1G>T
28	F	No	Hypotonia, PF/V, PW, hypertrichosis, DD, DR, encephalopathy, nystagmus	3	Alive 24 m	NA	NA	NA	Me, medial aspect of the cerebellar peduncles, Dt, Pu, Cp, PAG	Homo c.312_320del10insAT
29	F	Yes	PF, PW	birth	24 m	NA	NA	17% of LLR	NA	Homo c.799_800delCT
30	M	No	PW, DD, tremor, hypertrichosis. Post operative V, ophthalmoplegia, hypotonia	9 days	5y	NA	Low	NA	Ion, inferior pons, STh, Pu, spinocerebellar tracts, Icp, PAG, Dt, cerebellar WM	c.312_320del10insAT, c.871insT
31	F	No	PF/V, PW, DD, DR, tremor	1.5	3y 7 m	Normal	Low	NA	RN, Scp, PAG, Dt, deep cerebellar WM, Me, Ion, optic radiation	Homo c.792_793delAG
32	F	No	PW, DD, DR, PF, hypotonia	12	2y 3 m	Increased lipid	Low	NA	Mb, pons, Me, Icp, Dt, Cc, Cd, Pu, cerebellar WM, STh, SN,IC, long tracts in cervical spine	Homo c.754_755delAG
33	M	No	PF, PW, hypertrichosis, ataxia, DR, hypotonia, tremors, nystagmus, OA	14	7y 2 m	Reduced COX	Low	32% of LLR	Me, spinal cord, Pu, SN, IC, medullary pyramidal decussation, middle cerebellar peduncle	Homo c.312_320del10insAT
34	M	No	PW, PF/V, DD, DR, hypertrichosis, tremor, hypotonia, ophthalmoplegia, ataxia, RF, Sz	13	24 m	Absent COX increased lipid	Low	NA	Th, Mb, dorsal pons, Me	c.312_320del10insAT, c.574insCTGC
35	F	No	PW, PF/V, DD, DR, hypertrichosis, hypotonia, ophthalmoplegia, OA, nystagmus, tremor, ataxia, RF, Sz	9	2y 6 m	Absent COX, increased lipid	Post mortem	12% of LLR	Th	c.312_320del10insAT, c.751C >T
36	F	No	PW, PF/V, DD, DR, hypertrichosis, hypotonia, ophthalmoplegia, OA, nystagmus, tremor, ataxia, RF, Sz,	15	7y 10 m	Reduced COX, increased lipid	Post mortem	44% of LLR	CT brain: Pu	c.312_320del10insAT, c.688C>T
37	M	No	PF, ophthalmoplegia ataxia, hypotonia,Gross motor delay, PW, DR, DD, RF, Sz, hypertrichosis	birth	6y	Reduced COX	Low	NA	STh	c.312_320del10insAT, c. 752 -2A>G

*Age in months unless otherwise stated; a, b denote siblings; y/m years and months; NA not available.

Key to clinical and neuroimaging features: *BG* basal ganglia, *BS* Brain stem, *Cc* corpus callosum, *Cd* Caudate nucleus, *COX* cytochrome *c* oxidase, *Cp* cerebral peduncle, *Cst* corticospinal tracts, *DD* developmental delay, *DR* developmental regression, *Dt* Dentate, *Homo* homozygous, *GP* Globus pallidus, *IC* internal capsule, *Ico* Inferior colliculus, *Ion* inferior olivary nucleus, *Icp* inferior cerebellar peduncle, *LLR* lower limit of reference range, *Mb* Midbrain, *Me* Medulla, *NA* not available, *PAG* periaqueductal gray matter, *PF* poor feeding, *Pu* Putamen, *PW* poor weight gain, *RF* Respiratory failure, *RN* Red nucleus, *OA* optic atrophy, *Sco* superior colliculus, *Scp* superior cerebellar peduncle, *SN* Substantia Nigra, *STh* Subthalamic nucleus, *Sz* Seizures, *T1* type 1 muscle fibres, *T1FP* Type 1 muscle fibre predominance, *T2* type 2 muscle fibres, *Th* Thalamus, *UD* undetectable, *V* vomiting, *WM* white matter.

### Initial symptoms

The median age for the onset of first symptoms was 9.5 months (range 0–60 months) and the majority of the patients presented in the first year (32/44, 73%). The most frequently noted initial symptoms were poor feeding/vomiting (frequently attributed to gastro-oesophageal reflux) and poor weight gain (Table 2). The neonatal period was uneventful in the majority of patients (41/44, 93%) except in two patients with feeding problems and one patient with hypotonia. Developmental regression, defined as a loss of cognitive or motor skills, was the initial symptom in 3/44 (7%) patients. Most patients (26/44, 59%) presented with a combination of gastro-intestinal symptoms, poor weight gain and hypotonia. Three patients who underwent a general anaesthetic in the first two months experienced significant problems during the recovery period. One patient (case 30), who had surgery for transposition of the great arteries on day 9, developed persistent vomiting and required enteral feeding. The second patient (case 26) became hypotonic following surgery for pyloric stenosis at 8 weeks. The third patient (case 4) developed a movement disorder after an upper gastro-intestinal endoscopy.

**Table 2:** Initial symptoms in 44 patients with SURF1 deficiency

**Initial symptoms**	**Number of patients (%)**	**Age range of initial presentation (months)**
poor feeding/vomiting	20 (46)	0-24
poor weight gain	19 (43)	1.5-20
developmental delay	10 (23)	9-51
hypotonia	9 (21)	0-10
movement disorder	3 (7)	10-24
developmental regression	3 (7)	10-18
ataxia	2 (5)	14-60

### Major clinical features

**Figure 1:**
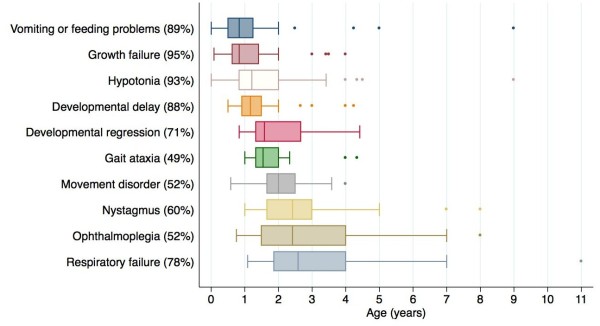
**Clinical features in 44 patients with SURF1 deficiency.** The x axis indicates the age of onset (years) and the y axis indicates the clinical features. Percentages denote the proportion of patients with a given clinical feature, and box-and-whisker plots show the age of onset. The median age of onset is indicated by the vertical line within the boxes. Boxes represent upper and lower quartiles, whiskers represent extreme values, and dots represent outliers which are ≥1.5 times the interquartile range from the median. Other less commonly observed features included hypertrichosis (41%), optic atrophy (23%), encephalopathy (20%), seizures (14%) and cardiomyopathy (2%).

Review of 44 cases with SURF1 deficiency revealed that 32 patients met the criteria for Leigh syndrome [3] and 12 patients were classified as “Leigh-like” due to atypical or normal radiological features or where neuroimaging was not available. The development of clinical features over time is demonstrated in Figure 1. Poor weight gain and hypotonia were the most prevalent symptoms, and tended to be preceded by gastro-intestinal symptoms such as poor feeding/vomiting (Figure 1). Long term enteral feeding was required in 21/44 (48%). Developmental regression (median age-of-onset 19 months) was noted in 27/38 (71%) cases and was precipitated by an intercurrent viral infection in 12 patients.

Hypertrichosis was reported in 18/44 (41%) patients. There was no significant correlation between the presence of hypertrichosis and lactic acidosis. Nystagmus and ophthalmoplegia were found in 25/42 (60%) and 22/42 (52%) patients, respectively, and occurred later (median age-of-onset 29 months). Movement disorder was noted in 22/42 (52%) patients (median age-of-onset 2 years) with an isolated intention tremor in 13 patients, choreoathetoid movements in 3, and dystonia in 2, while 4 patients demonstrated a combination of these abnormal movements. Only 6/44 (14%) patients reported seizures (generalised tonic clonic 5, myoclonic 1) and 4 of these patients were from Australia. Other less frequent clinical features included optic atrophy in 10/44 (23%), encephalopathy in 9/44 (20%) and hypertrophic cardiomyopathy in 1/44 (2%). None of the patients were reported to have pigmentary retinopathy or sensorineural deafness.

### Biochemical data

CSF lactate was elevated in all subjects who had this measured (mean 4.3, range 2.5-8.6 mmol/L, normal <2 mmol/L). Metabolic acidosis was present in 21/33 (64%) patients (mean bicarbonate level 14.7, range 10–17 mmol/L). 31/38 (81%) patients had elevated blood lactate (mean 4.4, range 2.3-7.3 mmol/L) (Table 3). Fibroblast COX activity was found to be low in all subjects who were tested (25/25, 100%) and ranged from levels below the detection limit of the assay, to 57% of the lower limit of the reference range (reference range 30–90 nmol/mg protein/min).

**Table 3:** Laboratory and magnetic resonance imaging (MRI) findings in SURF1 deficiency

**Laboratory or neuroimaging finding**	**Number of patients**	**%**
**Blood**		
Metabolic acidosis	21/33	64
Elevated lactate	31/38	81
**Cerebrospinal fluid**		
Elevated lactate	30/30	100
**Muscle**		
Reduced/absent COX histochemistry	23/33	70
Type I fibre predominance	8/33	24
Elevated muscle lipid content	16/33	48
Reduced muscle COX activity	25/26	96
**Fibroblast**		
Reduced fibroblast COX activity	25/25	100
**Nerve conduction studies**		
Peripheral neuropathy	13/16	81
**MRI lesions**		
Midbrain	12/33	36
Pons	10/33	30
Medulla	15/33	45
Putamen	16/33	48
Globus pallidus	14/33	42
Caudate nucleus	12/33	36
Subthalamic nucleus	4/33	12
Periaqueductal grey matter	4/33	12
Olivary nuclei	3/33	9
Red nuclei	2/33	6
Cerebellar white/grey matter	6/33	18
Cerebellar atrophy	3/33	9
Dentate nucleus	13/33	39
Cerebellar peduncles	8/33	24
Leukoencephalopathy	2/33	6

### Muscle biopsy

We analysed the histology and RCE findings of all patients where a muscle biopsy was performed (n = 33). Lipid content was increased in 15/33 (46%) of biopsies and type 1 fibre predominance was noted in 8 biopsies. COX histochemistry demonstrated absent COX in 9 (27%) biopsies and reduced COX staining in 14 (42%) biopsies. Ten (30%) of the biopsies were reported to have normal COX histochemistry with uniform staining of all fibres. RCE activities were measured in 28 biopsies. Two post-mortem biopsies were excluded since post-mortem delay may have resulted in artefactual loss of RCE activities. An isolated decrease of COX activity was found in all except one biopsy where the COX activity was at the lower end of the reference range (COX activity/citrate synthase ratio was 0.016 (reference range 0.014-0.034).

### Neuroimaging

Magnetic resonance imaging (MRI) and Computerised tomography scan (CT) information was available for 39 subjects (33 MRI and 6 CT scans). The patterns of involvement are shown in Table 3. Most MRIs showed findings characteristic of Leigh syndrome (28/33, 85%), with symmetrical hyperintense lesions on T2-weighted imaging in the brainstem and/or basal ganglia (Table 3). Two patients had normal MRI scans which were performed at 1 year of age and follow up imaging was not available. Two patients (one already published, [10]) had leukoencephalopathy (defined as a disorder that predominantly or exclusively affects the white matter of the brain [12]), while one patient had cerebellar atrophy and involvement of the dentate nucleus. Six patients had CT scans of which 3 were found to be normal and three had bilateral symmetric hypodensities in the basal ganglia.

### Peripheral nervous system

Sixteen patients had nerve conduction studies performed. Abnormal peripheral nerve conduction was noted in 13/16 (81%) with most (7) demonstrating a demyelinating neuropathy compared to an axonal neuropathy in 2 subjects. The type of neuropathy was unspecified in 4 subjects. Many (9/13, 69%) subjects showed a mixed sensori-motor involvement whereas 2 subjects had a pure sensory neuropathy.

### ***SURF1*** gene mutations

We identified *SURF1* mutations in 57 patients; 16/57(28%) had the homozygous c.312_320del10insAT insertion/deletion, 15/57(26%) were compound heterozygotes, 12/57(21%) had homozygous splice site mutations, 10/57(18%) had homozygous deletions, 2/57 (4%) had homozygous insertions, 1/57 (2%) had homozygous missense mutations and 1/57(2%) had homozygous nonsense mutations. The most frequently occurring mutation in our cohort was c.312_321del10insAT (p.Leu105X) (16 homozygous and 11 compound heterozygous). The next most common mutations were c.792_793delAG which occurred exclusively in Bangladesh Bengali subjects (n = 5, 3 pedigrees) and the splice mutation c.240+1G>T which was observed in 5 individuals, all of white European origin. The c.792_793delAG mutation has previously only been described in a patient of Japanese origin [13]. Individuals of Pakistani origin had the novel c.799_800delCT mutation or the c.324-11T>G mutation. The c.516-2A>G mutation segregated in two different Indian Gujarati pedigrees and has previously only been described in one subject of unspecified mixed Caucasian Asian parenthood [7]. The c.324-11T>G genotype was observed in 4 individuals from 2 different Pakistani pedigrees and one individual of Indian Gujarati origin. All five patients from the Australian cohort had at least one c.312_321del10insAT (p.Leu105X) mutation. No specific genotype phenotype correlation was observed. The patients surviving >10 years had varied *SURF1* genotypes: one was homozygous for the c.312_321del10insAT mutation, two were compound heterozygotes, two had homozygous insertions and one had a splice site mutation.

### Novel pathogenic mutations

**Figure 2:**
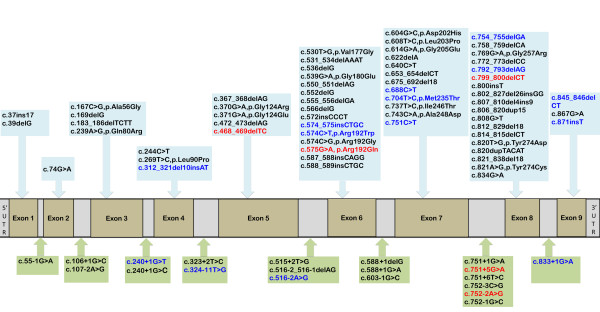
**Pathogenic mutations in the human *SURF1 *gene.** A schematic diagram of the *SURF1* gene illustrating 83 mutations reported to date. Red: novel unreported mutations identified in this study, blue: other mutations which have been previously reported and found in this study, black: mutations previously reported in the literature.

In our cohort we identified 19 *SURF1* mutations (Figure 2) in 57 patients including 5 novel pathogenic mutations (c.468_469delTC, c.799_800delCT, c.575G>A (p.Arg192Gln), c.751+5G>A. and c.752-2A>G).

Of the novel mutations identified, the homozygous c.799_800delCT was found in a Pakistani family where the patient (case 29) presented with early onset disease from birth and died at 2 years. The novel splice site c.751+5G>A mutation, generating an aberrant transcript with incorporation of 31 base pairs of intron 7 into a proportion of mature mRNAs, was found in one subject (case 3) who was a compound heterozygote (c.312_321del10insAT, c.751+5G>A). She had an atypical milder clinical course with longer survival. Her first concerning symptoms were at 16 months when she was noted to have mild motor delay and ataxia but in retrospect mild hypotonia was present from birth. She subsequently developed nystagmus and external ophthalmoplegia. She is currently studying business studies at 19 years of age. The novel missense mutation (c.575G>A, p.Arg192Gln) was found in a 12 year old female (case 2) with ataxia and a 4 year old male (case 22) with developmental delay, ataxia and hypertrophic cardiomyopathy. This was the only patient in the cohort with cardiomyopathy. The female patient subsequently developed nystagmus with ophthalmoplegia but she is currently able to ride a bicycle and play the violin. The splice site mutation c.752-2A>G was found in a patient (case 37) of Australian origin who presented with irritability and poor feeding since birth. Unfortunately clinical information was unavailable for the patient with the homozygous c.468_469delTC mutation.

### Survival

Among the 44 patients with detailed clinical data, five were alive at the time of writing (age range 2–19 years) while in three patients the current vital status was not known. Of the 36 deceased patients with known cause of death, the cause was central respiratory failure in 29/36 (80%). Seven patients survived beyond 10 years of age (cases 2, 3, 8a, 17, 20, 25a, 25b in Table 1). Of these, 6 presented with neurological symptoms such as ataxia and motor developmental delay; of note, gastro-intestinal symptoms were not the prominent presenting feature in these cases. Furthermore, these six patients also did not experience developmental regression.

**Figure 3:**
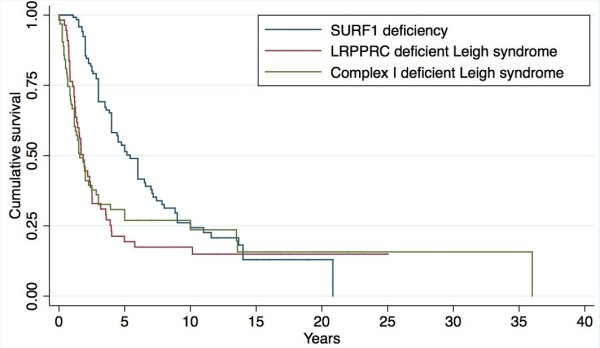
**Kaplan-Meier survival curves comparing SURF1 deficiency, LRPPRC-deficient LS and nuclear-encoded complex I deficient Leigh/Leigh-like syndrome.** Blue line: SURF1 deficient patients, n = 142 (this article and literature), red line: LRPPRC deficient patients, n = 56 [8], green line: complex I deficient Leigh/Leigh-like syndrome n = 63 [9]. Logrank test p < 0.001.

Literature searches identified 98 SURF1-deficient cases with available survival data, which we pooled together with the data from our 44 cases. In a Kaplan-Meier analysis (Figure 3), we compared the survival experience of these 142 SURF1-deficient cases to two other groups with LS due to nuclear gene mutations. We compared the survival of SURF1 deficiency to 56 patients with LRPPRC deficiency [8] and 63 patients with nuclear-encoded complex I-deficient LS/“Leigh- like” disease [9] Median survival length was longer in patients with SURF1 deficiency (median 5.4, 25th centile 3.0, 75th centile 10 years) than in patients with LRPPRC deficiency (median 1.8, 25th centile 1.0, 75th centile 4 years), and nuclear-encoded complex I-deficient LS (median 1.6, 25th centile 1.0, 75th centile 10 years) (p < 0.001 for difference across groups; logrank test).

### Additional cases in the literature

A systematic review of the literature revealed published clinical data on 129 SURF1-deficient patients and mutation data in a further 28 cases. The findings are summarised in Additional file 1: Table S1. Features which were found in these patients but not present in our cohort included unspecified hepatic involvement (3 cases), renal tubulopathy (4 cases) and facial dysmorphism (5 patients) [7,14-19].

## Discussion

SURF1 deficiency is the most common single cause of Leigh syndrome in the UK population, and careful documentation of the clinical features may lead to increased recognition and more rapid diagnosis of affected children. The need for early genetic diagnosis can be particularly pressing when affected families seek prenatal diagnosis. Improving the diagnostic process relies on well-recognised clinical features, but often there are inadequate numbers of patients with a single gene disorder of mitochondrial energy metabolism to characterise phenotypes. We have collected a cohort of 57 SURF1-deficient patients with comprehensive phenotypic data on 44 patients. We found that SURF1-deficient patients have a relatively homogenous phenotype which typically commences in late infancy with gastro-intestinal symptoms, followed by episodic neurological regression, ophthalmoplegia, movement disorder, finally leading to death from respiratory failure. Although there is a consistent clinical phenotype, there were no clinical characteristics that allowed discrimination of SURF1 deficiency from other causes of LS, such as complex I deficiency or complex V deficiency.

Cardiomyopathy was rare (one patient), and this observation is similar to the literature where only 2/129 cases had cardiomyopathy [14,18]. Seizure disorders also appeared to be uncommon and were found predominantly in the Australian patients which may indicate an environmental trigger which precipitates seizures. High temperature is unlikely to be the precipitating factor since no seasonal variation in seizures was observed in the cases from Australia. The low incidence of seizures is comparable with cases described previously where only 5% (7/129) were reported to have had seizures. Sensorineural deafness was not found in this SURF1-deficient cohort and is in keeping with the low occurrence in the literature where only two previous cases had sensorineural deafness [14]. Three (2%) cases were previously reported to have optic atrophy [6,18] whereas 10 (23%) of our patients were noted to have optic atrophy. Visual and auditory signs are difficult to ascertain in paediatric practice and may have been underdiagnosed in many cases where formal testing was not done. Three patients who underwent an early general anaesthetic experienced neurological deterioration; caution must be exercised when considering general anaesthesia for SURF1-deficient patients..

Although long-term survival in SURF1 deficiency is unusual, somewhat surprisingly we found that SURF1-deficient LS has a more favourable survival outcome compared to complex I-deficient LS or LRPPRC-deficient LS. Unlike SURF1 disease, LRPPRC deficiency is complicated by acute metabolic crises, which contribute significantly to the mortality [8].

Stepwise neurological regression often precipitated by illness and other states of high-energy demand, is thought to be part of the natural history of LS. In our cohort 6 patients surviving >10 years did not experience developmental regression which suggests that developing effective therapeutic manoeuvres to prevent and treat episodic decompensation could improve the outcome in SURF1-deficient patients. However the prevention of decompensation may be challenging in instances where an obvious precipitant cannot be identified. It is difficult to comment about the effect of supportive therapies in SURF1 deficiency since there is insufficient treatment data available for this cohort to draw any meaningful conclusions. Recent candidate therapies such as EPI-743 are under evaluation and improvement in quality of life and motor function scores have been demonstrated in 2 SURF1-deficient patients recruited to an open-label phase 2A study [20].

We describe 5 novel mutations in addition to the 78 mutations found in the *SURF1* gene so far. Approximately 80% of the mutations found in SURF1 deficiency are truncating mutations resulting from aberrant splicing, frameshift deletions or nonsense mutations. Our study shows segregation of specific mutations within certain ethnic groups. The genotype distribution suggests founder effects for some mutations such as c.311_312insATdel10 in white Europeans and c.790_800delAG demonstrated in 3 different Bangladesh Bengali pedigrees. Previous reports indicate that c.604G > C mutation has only been reported in Chinese individuals. We did not find a distinctive genotype-phenotype association although earlier reports [21] suggest missense *SURF1* mutations favour a better prognosis. It has not been possible to confirm this hypothesis in our cohort since only three subjects had missense mutations (1 homozygous, 2 compound heterozygous). However two of these patients are currently alive at 12 years and 17 years. The 7 patients described with milder phenotypes had varied genotypes including homozygosity for the common c.312_320del10insAT, suggesting that there are other factors which may influence survival.

We demonstrate that SURF1 deficiency has a distinct biochemical phenotype which includes consistently elevated CSF lactate, and decreased activities of COX in fibroblasts and muscle. Therefore when a typical clinical presentation of LS is encountered, if fibroblast COX activity is found to be low, targeted sequencing of the *SURF1* gene should be undertaken. If the patient is deteriorating rapidly it is prudent to perform a muscle biopsy with initial investigations since there are no distinctive clinical features of SURF1 deficiency which allow discrimination from other causes of LS. Muscle histochemistry shows uniform global reduction of COX activity but this may be very subtle and reported as normal since many centres use a prolonged incubation for COX histochemistry to allow maximum differentiation between COX-positive and negative fibres. Muscle histology demonstrated non-specific abnormalities such as lipid accumulation or fibre type disproportion (type I fibre predominance). Ragged red fibres which typically suggest a mitochondrial DNA mutation, have only been noted in two previous SURF1-deficient cases [14,18] and were not found in any patients in our cohort. The clinical picture encountered in SURF1 deficiency is typical for LS, and it is only with a supportive biochemical picture such as low fibroblast and/or muscle COX activity that SURF1 deficiency can be suspected.

Previous reviews indicate that involvement of the subthalamic nucleus was a consistent finding on MRI in SURF1 disease [22]. In our study only 4 (12%) had lesions in the subthalamic nucleus. In contrast 16/33 (48%) patients had lesions in the putamen. The specificity of these lesions in SURF1 deficiency needs to be validated by comparison to other groups with LS. Most (85%) MRIs showed characteristic lesions of LS. Leukoencephalopathic changes were an atypical finding (n = 2) but SURF1 deficiency should not be overlooked in these cases [10]. The MRI was normal in 2 subjects at 1 year of age. Normal neuroradiology early in the disease may subsequently evolve to abnormality on repeat imaging [18].

The presence of a demyelinating peripheral neuropathy has been noted in previous cases in the literature [23,24] and here we demonstrate that while in the majority of the cases the neuropathy is indeed of a demyelinating nature, an axonal neuropathy can be present.

Our study has several limitations. Although the study data were collected using a standardised questionnaire this is a retrospective study where clinical information was obtained by reviewing medical notes/case histories of patients, many now deceased. However it is unlikely that a large-scale prospective study will be conducted for this rare disease. The MRIs were performed at different centres and reviewed by different neuroradiologists which makes it difficult to ascribe specific patterns of disease. The MRI findings are based on a single scan in the majority of the cases and would only represent a snapshot of the neuroradiological changes but not disease progression. It is often not possible to capture these changes since repeated imaging in young children usually involves general anaesthetic with the attendant risks of metabolic decompensation.

## Conclusions

In conclusion, in contrast to other nuclear genes causing COX deficiency such as *SCO1*, *COX10* and *COX15*, SURF1 deficiency causes a largely homogeneous phenotype of LS characterised by systemic COX deficiency. To our knowledge, this is the first natural history study of SURF1 deficiency. Here we provide the most robust clinical description of the disease to date and a comprehensive account of all the genotypes and phenotypes reported so far. The prognostic and survival data in this study is based on a larger sample size than previous cohorts and will enable clinicians to be more confident when counselling families about the disease course. Advances in our understanding of the clinical spectrum and pathophysiology of SURF1 deficiency will be vital in planning therapeutic interventions in the future.

## Abbreviations

COX: Cytochrome *c* oxidase; CSF: Cerebrospinal fluid; CT: Computerised tomography; LS: Leigh Syndrome; MRI: Magnetic resonance imaging; OXPHOS: Oxidative phosphorylation.

## Competing interests

The authors declare that they have no competing interests.

## Authors’ contributions

YW participated in study design, acquired data, coordinated data collection, analysed data, interpreted data and wrote manuscript. RB performed molecular genetic studies, interpreted data and revised manuscript. GKB was involved in study supervision, study design, revising manuscript, and interpretation of data. ETA analysed data, performed statistical analysis and produced figures. RM, JYL, AAM, MC, PEJ, AC, DRT, RWT, JML, KF, AD, LS, DK, DH and AC acquired and interpreted data. SR conceived the idea for the study, designed study, supervised study, interpreted data, drafted and revised manuscript content. All authors read and approved the final manuscript.

## Authors’ information

This work was supported by the following grants: YW is supported by a Wellcome Trust Research Training Fellowship (grant number 097978/Z/11/Z) and SR is supported by a Great Ormond Street Hospital Children’s Charity Research Leadership Grant.

## Supplementary Material

Additional file 1 SURF1 study data collection questionnaire and review of cases in the literature. Click here for file
