# Supplementary material for: SURF1 deficiency: a multi-centre natural history study
Source: Orphanet J Rare Dis. 2013 Jul 5;8:96. doi: 10.1186/1750-1172-8-96 (PMC3706230; doi:10.1186/1750-1172-8-96)
Supplement: Additional file 1 — SURF1 study data collection questionnaire and review of cases in the literature. [file 1750-1172-8-96-S1.docx]

| **Additional material 1: SURF1 study data collection questionnaire** | |
| --- | --- |
|  |  |
|  |  |
| Name of hospital |  |
| Speciality of managing consultant (Metabolic, Neurology, General Paediatric or other specify) | Metabolic  Neurology General Paediatric  other specify |

**Patient demographics**

| Patient |  |
| --- | --- |
| Date of birth |  |
| Ethnicity |  |
| Gender |  |
| Is the patient alive or deceased? |  |
| If deceased, please give age or date of death. |  |
| If deceased, please give cause of death. |  |

**Family history**

| Is there a history of consanguinity? |  |
| --- | --- |
| If so, degree of relationship |  |
| Does the patient have any affected siblings?  Please give sex and ages of affected siblings. |  |
| **Pedigree** |  |

**Prenatal history:**

| **Prenatal and birth history** | **Details** |
| --- | --- |
| Problems noted during pregnancy of the affected child? |  |
| Peri partum issues? |  |
| Mode of delivery and gestational age at the time of birth. |  |
| Birth weight and centile |  |
| Birth length and centile |  |
| Birth head circumference and centile |  |

**Clinical Features**

| **Clinical Diagnosis** | **Tick as appropriate** |
| --- | --- |
| Leigh Syndrome |  |
| Leukoencephalopathy |  |
| Other (please specify) |  |

| **Initial symptoms and age at first presentation** |  |
| --- | --- |

**Subsequent symptoms and age of onset**

| **Clinical feature** | **Yes/ No** | **If yes, age of onset** | **Other information** |
| --- | --- | --- | --- |
| Poor feeding or vomiting? |  |  |  |
| Evidence of poor growth? |  |  | Weight centile      at age  Height centile       at age |
| Hypotonia or floppy? |  |  |  |

| **Clinical feature** | **Yes/**  **No** | **If yes, age of onset** | **Other information** |
| --- | --- | --- | --- |
| Movement disorder (e.g. tremor, chorea etc) |  |  | Please specify type |
| Ataxia? |  |  |  |
| Developmental delay? |  |  | Motor Speech  Intellectual Global |
| Developmental regression? |  |  | Any triggering factors? |
| Encephalopathy? |  |  |  |
| Seizures? |  |  | Generalised tonic clonic Myoclonic Absence seizures Other  Specify |
| Nystagmus? |  |  | Horizontal  Vertical  Rotatory |
| Ptosis or ophthalmoplegia? |  |  |  |
| Optic atrophy? |  |  |  |
| Pigmentary retinopathy? |  |  |  |
| Sensorineural hearing loss? |  |  |  |
| Peripheral neuropathy? |  |  |  |
| Hypertrichosis? |  |  |  |
| Dermatological problems? |  |  |  |
| Respiratory failure? |  |  |  |
| Any other symptoms or signs not mentioned above? |  |  |  |
| Age at definitive diagnosis? |  |  |  |
| Number of PICU admissions |  |  | Cause for each admission |

**Other information (free text)**

| Is the patient in mainstream school? Please give details (special needs etc) |
| --- |
|  |
| Does the patient require any specific aids? e.g. wheelchair. Please give age at which these were first required |
|  |

**Imaging and EEG findings**: **if you have an electronic version of the report please copy and paste**

| **Investigation** | **Date** | **Result** |
| --- | --- | --- |
| MRI brain scan 1 |  |  |
| MRI brain scan 2 (if more than one scan) |  |  |
| MRI brain scan 3 (if more than two scans) |  |  |
| EEG findings |  |  |
| Echocardiogram (cardiac) findings  Cardiomyopathy? (If so, hypertrophic or dilated?) |  |  |
| ECG abnormality (Conduction defect?) |  |  |
| Nerve conduction studies/ EMG |  |  |

**Treatment**

| **Treatment** | **Yes/No** | **Details** |
| --- | --- | --- |
| Coenzyme Q_10_ (Ubiquinone) |  |  |
| Other vitamins?  Singly or in cocktail? |  |  |
| Naso-gastric or PEG feeding (please give age of commencement) |  |  |

**Investigations: (if you have an electronic version of the report please copy and paste or attach copy)**

1. **Muscle biopsy**

| Ragged red fibres | SDH-positive fibres | COX-negative fibres (if so, patchy or homogeneous) | Ragged blue fibres (on COX/SDH stain) | Excess lipid | Electron microscopy changes | Other muscle histological abnormalities |
| --- | --- | --- | --- | --- | --- | --- |
|  |  |  |  |  |  |  |

1. **Respiratory chain enzyme activities: Please give reference ranges for each item.**

| **Enzyme activity** | **In muscle** | **Ref range** | **In liver** | **Ref range** | **In fibroblasts** | **Ref range** |
| --- | --- | --- | --- | --- | --- | --- |
| Complex I |  |  |  |  |  |  |
| Complex II+III |  |  |  |  |  |  |
| Complex IV |  |  |  |  |  |  |
| Complex V |  |  |  |  |  |  |

|  | muscle | white cell |
| --- | --- | --- |
| Coenzyme Q_10_ level |  |  |

1. **Other metabolic investigations. Please include interpretive comments (e.g. for organic acid results) provided by the lab and local reference ranges**

| **Investigation** | **Date of investigation** | **Result (with units)** | **Reference range** |
| --- | --- | --- | --- |
| Serum lactate |  |  |  |
| Serum pyruvate |  |  |  |
| Serum lactate/pyruvate ratio |  |  |  |
| If serum lactate was done more than once please give range (e.g from 2.1 to 4 mmol/L) |  |  |  |
| Metabolic acidosis? If yes, please give pH & HCO_3_ below |  |  |  |
| pH |  |  |  |
| HCO_3_ |  |  |  |
| Plasma amino acids (PAA)   - Alanine - Other abnormalities in PAA |  |  |  |
| Blood spot or plasma acylcarnitines |  |  |  |
| Hypoglycaemia? If yes give result |  |  |  |
| Ketosis? |  |  |  |
| Urine organic acids |  |  |  |
| CSF amino acids   - Alanine - Other abnormalities |  |  |  |
| CSF protein |  |  |  |
| CSF lactate |  |  |  |
| CSF pyruvate |  |  |  |
| CSF lactate/pyruvate ratio |  |  |  |
| CSF neurotransmitters |  |  |  |
| Renal tubular function  Urine retinol binding protein/creatinine RBP/Cr ratio |  |  |  |
| Renal tubular function  Urine N-acetylglucosaminidase NAG/Cr ratio |  |  |  |
| Endocrine disturbance? Please specify |  |  |  |

**Any other relevant information not indicated above**

Additional table 1: Patient phenotypes and genotypes published in the literature

|  | **Age of onset** | **Age of death** | **Clinical features** | **MRI** | **Blood lactate** | **CSF lactate** | **Histology** | **COX histo-chemistry** | **Muscle COX** | **Fibro-blast COX** | **Mutation allele 1*** | **Mutation allele 2*** | **Reference** |
| --- | --- | --- | --- | --- | --- | --- | --- | --- | --- | --- | --- | --- | --- |
| 1 | NA | NA | Hypotonia, truncal ataxia, ophthalmoplegia, psychomotor regression, respiratory failure | NA | NA | NA | NA | NA | low | low | c.37ins17 | c.37ins17 | Tiranti et al 1998 |
| 2 | NA | NA | Hypotonia, truncal ataxia, ophthalmoplegia, psychomotor regression, respiratory failure | NA | NA | NA | NA | NA | low | low | c.550delAG | c.516+2 | Tiranti et al 1998 |
| 3 | NA | NA | Hypotonia, truncal ataxia, ophthalmoplegia, psychomotor regression, respiratory failure | NA | NA | NA | NA | NA | low | low | c.868insT | c.868insT | Tiranti et al 1998 |
| 4 | NA | NA | Hypotonia, truncal ataxia, ophthalmoplegia, psychomotor regression, respiratory failure | NA | NA | NA | NA | NA | low | low | c.312_321del10insAT | c.312_321del10insAT | Tiranti et al 1998 |
| 5 | NA | NA | Hypotonia, truncal ataxia, ophthalmoplegia, psychomotor regression, respiratory failure | NA | NA | NA | NA | NA | low | low | c.845delCT | c.845delCT | Tiranti et al 1998 |
| 6 | NA | NA | Hypotonia, truncal ataxia, ophthalmoplegia, psychomotor regression, respiratory failure | NA | NA | NA | NA | NA | low | low | c.845delCT | c.312_321del10insAT | Tiranti et al 1998 |
| 7 | NA | NA | Hypotonia, truncal ataxia, ophthalmoplegia, psychomotor regression, respiratory failure | NA | NA | NA | NA | NA | low | low | c.751C>T | c.751C>T | Tiranti et al 1998 |
| 8 | NA | NA | Hypotonia, truncal ataxia, ophthalmoplegia, psychomotor regression, respiratory failure | NA | NA | NA | NA | NA | low | low | c.845delCT | c.845delCT | Tiranti et al 1998 |
| 9 | NA | NA | Hypotonia, truncal ataxia, ophthalmoplegia, psychomotor regression, respiratory failure | NA | NA | NA | NA | NA | low | low | c.772delCC | c.772delCC | Tiranti et al 1998 |
| 10 | NA | NA | NA | NA | NA | NA | NA | NA | NA | NA | c.751C>T | c.323+2T>C | Zhu et al 1998 |
| 11 | NA | NA | NA | NA | NA | NA | NA | NA | NA | NA | c.845_846delCT | c.312_321del10insAT | Zhu et al 1998 |
| 12 | NA | NA | NA | NA | NA | NA | NA | NA | NA | NA | c.868insT | c.868insT | Zhu et al 1998 |
| 13 | 16 months | 3 years | Typical LS | NA | LS | NA | NA | NA | low | low | c.751C>T | c.751C>T | Tiranti et al 1999 |
| 14 | 12 months | 3 years | Typical LS | NA | LS | NA | NA | NA | low | low | c.588insCTGC | c.74G>A | Tiranti et al 1999 |
| 15 | 11 months | 4 years | Typical LS | NA | LS | NA | NA | NA | low | low | c.552delG | c.552delG | Tiranti et al 1999 |
| 16 | 12 months | 4 years | Typical LS | NA | LS | NA | NA | NA | low | low | c.312del10,insAT | c.312del10,insAT | Tiranti et al 1999 |
| 17 | 12 months | 4 years | Typical LS | NA | LS | NA | NA | NA | NA | low | c.312del10,insAT | c.845delCT | Tiranti et al 1999 |
| 18 | 7 months | 6 years | Typical LS | NA | LS | NA | NA | NA | NA | low | c.37ins17 | c.37ins17 | Tiranti et al 1999 |
| 19 | 10 months | 2 years | Typical LS | NA | LS | NA | NA | NA | NA | low | c.868insT | c.868insT | Tiranti et al 1999 |
| 20 | 6 months | alive at 8 years | Typical LS | NA | LS | NA | NA | NA | low | low | c.845delCT | c.845delCT | Tiranti et al 1999 |
| 21 | 9 months | died at 6 years | Typical LS | NA | LS | NA | NA | NA | low | low | c.552delG | c.552delG | Tiranti et al 1999 |
| 22 | 9 months | 7 years | Typical LS | NA | LS | NA | NA | NA | low | low | c.312_321del10insAT (p.L105X) | c.822G>T | Tiranti et al 1999 |
| 23 | 13 months | 3 years | Typical LS | NA | LS | NA | NA | NA | low | low | c.588insCTGC | c.588insCTGC | Tiranti et al 1999 |
| 24 | 5 months | alive 4 year | Typical LS | NA | LS | NA | NA | NA | low | NA | c.758del2bp | c.758del2bp | Tiranti et al 1999 |
| 25 | 16 months | 3 years | Typical LS | NA | LS | NA | NA | NA | low | low | c.516+2T>G | c.552delG | Tiranti et al 1999 |
| 26 | 12 months | 9 years | Typical LS | NA | LS | NA | NA | NA | low | low | c.239+1T>G | c.239+1T>G | Tiranti et al 1999 |
| 27 | 14 months | 3 years | Typical LS | NA | LS | NA | NA | NA | low | low | c.312_321del10insAT (p.L105X) | c.312_321del10insAT (p.L105X) | Tiranti et al 1999 |
| 28 | 8 months | 3 years | Typical LS | NA | LS | NA | NA | NA | low | NA | c.845delCT | c.845delCT | Tiranti et al 1999 |
| 29 | 12 months | 8 years | Typical LS | NA | LS | NA | NA | NA | low | NA | c.845delCT | c.845delCT | Tiranti et al 1999 |
| 30 | Died | 14 years | Typical LS | NA | LS | NA | NA | NA | low | low | c.772_773delCC | c.772_773delCC | Tiranti et al 1999 |
| 31 | 10 months | alive at 18 months | Motor developmental delay, ophthalmoplegia, respiratory failure | Bilateral, symmetrical signal increases in basal ganglia, cerebellum dentate nucleus, and around aqueduct of midbrain | normal | high | Variation in fibre size | low | NA | NA | c.790delAG | c.820C>G | Teraoka et al1999 |
| 32 | shortly after birth | 6 years | Developmental delay, short stature, hypertrichosis, intention tremor, hypotonia | Bilateral and symmetric areas of lucencies in the brainstem and cerebellum | high | NA | Variation in fibre size | low | low | NA | c.258C>T | c.258C>T | Santoro et al 2000 |
| 33 | NA | NA | NA | NA | NA | NA | NA | NA | NA | NA | c.312_321del10insAT | c.587_588insCAGG | Sue et al. 2000 |
| 34 | NA | NA | NA | NA | NA | NA | NA | NA | NA | NA | c.845_846delCT | c.814delCT | Sue et al. 2000 |
| 35 | NA | NA | NA | NA | NA | NA | NA | NA | NA | NA | c.845_846delCT | c.845_846delCT | Sue et al. 2000 |
| 36 | 2 years | alive at 7 years | Tremor, cerebellar ataxia, deafness, dystonia, ophthalmoplegia, encephalopathy | NA | NA | NA | NA | NA | NA | NA | G618 C, T751 C | c.766–3C>G | Poyau et al 2000 |
| 37 | 14 months | 3 years | Hypotonia, abnormal involuntary movements, tremor, ataxia, deafness, partial Fanconi syndrome, severe encephalopathy, cardiomyopathy | NA | high | NA | NA | NA | NA | NA | c.702C>T | c.589ins CTGC | Poyau et al 2000 |
| 38 | 4 years | alive at 4 years | Short stature, psychomotor regression, muscular atrophy, hypotonia, encephalopathy | NA | NA | NA | Ragged red fibres | low | NA | NA | c.312_321del10insAT | c.385G>A e | Poyau et al 2000 |
| 39 | 3 years | alive at 3 years | NA | NA | NA | NA | NA | NA | NA | NA | c.312_321del10insAT | c.385G>A e | Poyau et al 2000 |
| 40 | 2 months |  | Vomiting , hypotonia, developmental delay, poor growth, nystagmus, tremors | High intensity in lenticular nucleus | high | NA | NA | low | NA | N\|A | Homozygous insertion of one base in exon 9 | Homozygous insertion of one base in exon | Savasta et al 2001 |
| 41 | 9 months | 4 years | Poor growth, swallowing difficulties, hypotonia, trunk ataxia, nystagmus, optic atrophy, demyelinating peripheral neuropathy | Symmetric lesions of basal ganglia and brain stem | high | NA | NA | NA | low | NA | c.312_321del10insAT | c.588+1delG | Pe´quignot et al 2001 |
| 42 | NA | alive at 14 years | Psychomotor delay, ataxia, ophthalmoplegia, retinopathy, demyelinating neuropathy, recurrent vomiting attacks and regression | Brain stem, thalamus and cerebellar atrophy | high | NA | NA | NA | low | NA | c.312_321del10insAT | c.737T>C | Pe´quignot et al 2001 |
| 43 | 12 months | 2 years | Poor growth, trunk ataxia, dystonia, swallowing difficulties in swallowing, respiratory distress, optic atrophy, demyelinating neuropathy | Bilateral symmetrical lesions of the basal ganglia and the brain stem | high | NA | NA | NA | low | NA | c.240+1G >T | c.588+1G>A | Pe´quignot et al 2001 |
| 44 | NA | 18 months | Vomiting, developmental delay, hypotonia | NA | NA | NA | NA | NA | low | NA | c.516–2_516–1delAG | c.516–2_516–1delAG | Pe´quignot et al 2001 |
| 45 | 6 months | 34 months | Poor growth, hypertrichosis, ophthalmoplegia, hypotonia, severe cerebellar disturbances | Symmetrical lesions of the basal ganglia | high | high | NA | NA | low | low | c.312_321del10insAT | c.821del18 | Williams et al 2001 |
| 46 | birth | 6 years | Hypotonia, muscle weakness, ataxia, dystonia, choreoathetosis, poor growth, hypertrichosis, hepatopathy | NA | high | high | NA | NA | low | NA | c.688C>T | c.820-821dup | Darin et al 2001 |
| 47 | 12 months | alive at 1 year | Hypotonia, muscle weakness, ataxia, dystonia, choeoathetoiss, poor growth,, ophthalmoplegia | NA | high | high | NA | NA | low | NA | c.312_321del10insAT | c.312_321del10insAT | Darin et al 2001 |
| 48 | 5 months | alive at 5 months | Nystagmus, hypotonia, neuropathy, poor growth | NA | high | high | NA | NA | low | NA | c.688C>T | C.751+1G>A | Darin et al 2001 |
| 49 | 6 months | 3 years | Sudden poor growth, vomiting, major hypertrichosis, ophthalmoplegia, hypotonia | CT scan was normal | high | NA | NA | NA | low | low | c.539G>A, p.G160E | c.603-1G>C | Von Kleist Retzow et al 2001 |
| 50 | NA | NA | NA | NA | NA | NA | NA | NA | NA | NA | c.367_368delAG | c.367_368delAG | Ogawa et al 2002 |
| 51 | 12-15 months | alive at 15 months | Poor feeding, short stature, muscle weakness, movement disorder, liver involvement, hypertrichosis | Bilateral hyperintense lesions in putamen, the subcortical white matter, cerebral peduncles, pons, medulla oblongata, nucleus dentatus | NA | NA | NA | NA | low | NA | c.312_321del10insAT | c.312_321del10insAT | Moslemi et al 2003 |
| 52 | 5 months | alive at 5 months | Poor feeding, short stature, muscle weakness, movement disorder, nystagmus, external ophthalmoplegia | Hyperintense lesions on T2-weighted imaging in putamen, the cerebral peduncles, pons, and around the aqueductus cerebri. | NA | NA | NA | NA | low | NA | c.688C>T | tandem duplication (806–820) in exon 8, | Moslemi et al 2003 |
| 53 | 5 months | NA | Poor feeding, short stature, nystagmus | NA | NA | NA | NA | NA | low | NA | c.688C>T | C.751+1G>A | Moslemi et al 2003 |
| 54 | NA | NA | Poor growth, progressive muscle hypotonia, hypertrichosis, developmental delay, amaurosis | Symmetrical basal ganglia lesions | NA | NA | NA | NA | NA | low | c.841delCT | c.841delCT | Pecina et al 2003 |
| 55 | NA | NA | Poor growth, progressive muscle hypotonia, hypertrichosis, developmental delay, ophthalmoplegia | Symmetrical basal ganglia lesions | NA | NA | NA | NA | NA | low | c.312_321del10insAT | c.821del18 | Pecina et al 2003 |
| 56 | NA | alive at 11 years | Poor growth, progressive muscle hypotonia, hypertrichosis, developmental delay, nystagmus | symmetrical basal ganglia lesions | NA | NA | NA | NA | NA | low | 841delCT mutation | c.574C>T | Pecina et al 2003 |
| 57 | 14 months | 22 months | Developmental delay, hypotonia, regression, peripheral neuropathy, respiratory failure | Brain MRI at 16 months: showed bilateral, symmetric lesions involving the medulla above, at, and below the pyramidal decussation and symmetrically the inferior cerebellar peduncles, substantia nigra, central tegmental tracts, and subthalamic nuclei | high | NA | NA | absent | low | NA | c.240+1G >T | c.531_534delAAAT | Rossi et al 2003 |
| 58 | 12 months | alive at 3 years | Poor growth, neurodevelopmental regression, frequent vomiting, hypertrichosis, hypotonia | Brain MRI: T2 prolongation involving the medulla above, at, and below the pyramidal decussation and extending to the cervical spinal cord, inferior cerebellar peduncles, dentate nuclei, substantia nigra, and subthalamic nuclei | high | NA | NA | absent | low | NA | c.566delG | mutation at splice-junction site of intron 4 | Rossi et al 2003 |
| 59 | 1 st year | alive at 4 years | Poor growth, neurodevelopmental delay, palpebral ptosis, impaired consciousness, facial dysmorphism, hirsutism, nystagmus, hypotonia, respiratory abnormalities. | Brain MR imaging was performed at age 3 years: medulla, inferior cerebellar peduncles, dentate nuclei, midbrain, substantia nigra, central tegmental tract, interpeduncular nucleus, pallido-cortical–nigro-cortical tracts. subthalamic nuclei, putamina | high | NA | NA | absent | low | NA | c.772_773delCC | c.772_773delCC | Rossi et al 2003 |
| 60 | NA | NA | Poor growth, progressive muscle hypotonia, hypertrichosis, developmental delay, ophthalmoplegia | Symmetrical basal ganglia lesions | NA | NA | NA | NA | NA | low | c.688C>T, | c.688C>T | Pecina et al 2003 |
| 61 | 4 months | alive at 8 years | Poor growth, developmental delay, ataxia, nystagmus, tremors, dysphagia, respiratory failure | NA | NA | NA | NA | NA | low | NA | c.312_321del10insAT | c.572_573insCCCT | Salviati et al 2004 |
| 62 | NA | NA | Poor feeding, hypotonia, poor growth, developmental delay, respiratory abnormalities | Neuroradiological changes of Leigh syndrome on neuroimaging | high | NA | NA | NA | NA | NA | c.312_321del10insAT |  | Head et al 2004 |
| 63 | NA | NA | Poor feeding, hypotonia, poor growth, developmental delay, respiratory abnormalities | Neuroradiological changes of Leigh syndrome on neuroimaging | high | NA | NA | NA | NA | NA | c.312_321del10insAT |  | Head et al 2004 |
| 64 | NA | NA | Poor feeding, hypotonia, poor growth, developmental delay, respiratory abnormalities | Evidence only of cerebral atrophy, particularly affecting the frontal and parietal cortex | normal | high | NA | NA | NA | NA |  |  | Head et al 2004 |
| 65 | 4 months | alive at 42 months | Poor growth, psychomotor developmental delay, nystagmus, ataxia tremor, dysphagia, apnoea | First MRI brain at 39 months normal. Second study at 8 years: diffuse supratentorial atrophy, severe cerebellar hemisphere atrophy, and cystic-like changes inferior to pontine brainstem. White matter signal intensity in supratentorial region normal and basal ganglia also appeared normal | normal | NA | NA | NA | NA | NA | c.312_321del10insAT | c.572_573insCCCT; | Salviati et al 2004 |
| 66 | 6 months | alive at 11 months | Persistent vomiting, poor growth, developmental regression, cardiomyopathy, respiratory failure, distal tubular acidosis | 8 months: symmetric bilateral signal abnormalities throughout brain stem, cerebellum, and diencephalon and a remote posterior inferior cerebellar artery infarction | high | high | NA | low | NA | NA | c.312_321del10insAT | c.688C>T | Tay et al 2005 |
| 67 | 10 months | 2 years | Developmental regression, hypotonia, nystagmus, optic atrophy, bilateral Babinski reflexes, respiratory abnormalities, proximal tubular acidosis | Right and left putamen globus pallidus showed oedema | high | NA | Type 1 fibre atrophy | low | low | low | c.834G>A | c.834G>A | Tay et al 2005 |
| 68 | 3 months | 2 years | Loss of head control, poor growth, truncal ataxia, hypertrichosis, respiratory abnormalities | Midbrain tegmentum, brain stem, dentate nucleus. | high | NA | NA | low | low | low | c.834G>A | c.834G>A | Tay et al 2005 |
| 69 | 10 months | alive at 10 months | Vomiting, regression, motor delay, tubular acidosis | Bilateral abnormal signals in the putamen and caudate nuclei | normal | high | Increased lipid and ragged red fibres | low | low | low | c.312_321del10insAT | c.820–824dupTACAT | Tay et al 2005 |
| 70 | 2 months | 2 year and 7 months | poor growth, progressive developmental delay, muscle weakness, hypertrichosis, squint | 2½ years: symmetric bilateral hyperintense lesions at T2, dentate nuclei, mesencephalic nuclei, periaqueductal parenchyma, and medulla oblongata | high | NA | NA | NA | low | low | c.312_321del10insAT | c.845delCT | Østergaard et al 2005 |
| 71 | 6 weeks |  | Psychomotor development delay, hypotonia, ataxia, dystonia ataxia, hypertrichosis, gastrostomy feeding | CT scan: symmetrical lesions in basal ganglia | NA | NA | NA | NA | low | low | c.312_321del10insAT | c.688C>T | Østergaard et al 2005 |
| 72 | 10 months | 9 years | Psychomotor developmental delay, poor growth, ophthalmoplegia, ataxia, spasticity, hypertrichosis | NA | NA | NA | NA | NA | low | low | c.312_321del10insAT | c.312_321delTCTGCCAGCCinsAT | Østergaard et al 2005 |
| 73 | NA | NA | NA | NA | NA | NA | NA | NA | NA | NA | c.240+1G>C, | NA | Yang et al 2006 |
| 74 | NA | NA | NA | NA | NA | NA | NA | NA | NA | NA | c.574C>G, | NA | Yang et al 2006 |
| 75 | NA | NA | NA | NA | NA | NA | NA | NA | NA | NA | c.622delA | NA | Yang et al 2006 |
| 76 | NA | NA | NA | NA | NA | NA | NA | NA | NA | NA | c.653-654delCT | NA | Yang et al 2006 |
| 77 | 23 months | alive at 23 months | regression, poor growth, hypotonia | 2 years: hypointense lesion of subthalamic nuclei, brainstem involvement; second MRI at 3 years: mild cerebellar and marked cerebral atrophy, diffuse involvement of substantia nigra, hyperintense lesion of central tegmental tract, and extensive brainstem involvement | NA | high | NA | NA | NA | NA | c.244C>T | c.244C>T | Yüksel et al 2006 |
| 78 | 13 months | alive at 3 years | Psychomotor regression, developmental delay, failure to thrive, neurodevelopmental regression, generalized hypotonia, respiratory problems, and absent deep facial dysmorphism including frontal bossing, brachycephaly, hypertrichosis | Heavy brainstem and subthalamic nuclei involvement without lesions in basal ganglia; second MRI at 2 years indicated heavy brainstem and subthalamic nuclei involvement without lesions in basal ganglia | NA | high | NA | NA | low | low | c.530T>G | c.530T>G | Yüksel et al 2006 |
| 79 | NA | NA | NA | NA | NA | NA | NA | NA | NA | NA | c.845-846delCT | c.845-846delCT | Bohm et al 2006 |
| 80 | NA | NA | NA | NA | NA | NA | NA | NA | NA | NA | c.845-846delCT | c.845-846delCT | Bohm et al 2006 |
| 81 | NA | NA | NA | NA | NA | NA | NA | NA | NA | NA | c.845-846delCT | c.312_321del10insAT | Bohm et al 2006 |
| 82 | NA | NA | NA | NA | NA | NA | NA | NA | NA | NA | c.845-846delCT | c.574C>T | Bohm et al 2006 |
| 83 | NA | NA | NA | NA | NA | NA | NA | NA | NA | NA | c.845_846delCT | c.756delCA | Bohm et al 2006 |
| 84 | NA | NA | NA | NA | NA | NA | NA | NA | NA | NA | c.845_846delCT | c.704T>C | Bohm et al 2006 |
| 85 | NA | NA | NA | NA | NA | NA | NA | NA | NA | NA | c.845_846delCT | c.821A>G | Bohm et al 2006 |
| 86 | NA | NA | NA | NA | NA | NA | NA | NA | NA | NA | c.821-838del18 | c.821-838del18 | Bohm et al 2006, |
| 87 | NA | NA | NA | NA | NA | NA | NA | NA | NA | NA | c.312_321del10insAT | c.821-838del18 | Bohm et al 2006 |
| 88 | NA | NA | NA | NA | NA | NA | NA | NA | NA | NA | c.312_321del10insAT | c.312_321del10insAT | Bohm et al 2006 |
| 89 | NA | NA | NA | NA | NA | NA | NA | NA | NA | NA | c.688C>T | c.688C>T | Bohm et al 2006 |
| 90 | 2 years | 6 years | ataxia, hypotonia, nystagmus | typical of leigh | high | high | NA | NA | low | low | c.370G>A | c.370G>A | Coenen et al 2006 |
| 91 | NA | NA | Leigh | NA | NA | NA | NA | NA | NA | low | C.244C>T | c.312_321del10insAT | Coenen et al 2006 |
| 92 | NA | NA | Leigh | NA | NA | NA | NA | NA | NA | low | c.239G>A | c.312_321del10insAT | Coenen et al 2006 |
| 93 | NA | NA | Leigh | NA | NA | NA | NA | NA | NA | low | c.312_321del10insAT | c.312_321del10insAT | Coenen et al 2006 |
| 94 | 12 months | 5 years | Poor growth, developmental delay, microcephaly. hypertrichosis, synophrys, hypotonia, peripheral neuropathy and truncal ataxia | Lesions in caudal parts of brain stem resulting in distension of medulla oblongata and lesions in central myelin of cerebellum | high | high | normal | normal | low | low | c.867G>A | c.867G>A | Van Riesan et al 2008 |
| 95 | 4 months | alive at 4 months | Motor regression, hypotonia, hypertrichosis, bulbar symptoms | NA | high | NA | NA | NA | low | NA | c.841delCT | NA | Pronicki et al 2008 |
| 96 | 12 months | alive at 12 months | Trembling, ataxia gait, myoclonic jerks, ophthalmoplegia | Hyperintense signals at lenticular nuclei | NA | NA | NA | NA | low | NA | c.841delCT | NA | Pronicki et al 2008 |
| 97 | 30 months | alive at 30 months | Dystonic movements, poor growth | Symmetric hyperintense signals at lenticular and caudate nuclei, medulla oblongata putamen, globi pallidi | NA | NA | NA | NA | low | NA | c.841delCT | NA | Pronicki et al 2008 |
| 98 | 5 months | alive at 5 months | Poor growth, hypotonia, nystagmus, trembling, hypertrichosis, respiratory failure | NA | NA | NA | NA | NA | low | NA | c.841delCT | C.841delCT | Pronicki et al 2008 |
| 99 | 12 months | 4 years | Poor growth, vomiting, respiratory abnormalities | Symmetric hypodense changes in basal ganglia | NA | NA | NA | NA | NA | NA | c.841delCT | C.841delCT | Pronicki et al 2008 |
| 100 | 16 months | alive at 16 months | Speech difficulties, hypotonia, nystagmus, developmental regression. | NA | NA | NA | NA | NA | low | NA | c.841delCT | NA | Pronicki et al 2008 |
| 101 | 2 years | Alive at 2 years | Difficulty in walking and speaking, poor growth, ophthalmoplegia, hypertrichosis, respiratory abnormalities | NA | NA | NA | NA | NA | low | NA | c.841delCT | C.841delCT | Pronicki et al 2008 |
| 102 | 12 months | alive at 12 months | Poor growth, hypotonia, hypertrichosis, respiratory abnormalities | NA | NA | NA | NA | NA | NA | NA | c.841delCT | C.841delCT | Pronicki et al 2008 |
| 103 | 14 months | 30 months | 14 months: regression of motor skills, poor growth, hypertrichosis, tremor, ophthalmoplegia, respiratory abnormalities | NA | NA | NA | NA | NA | low | NA | c.841delCT | C.841delCT | Pronicki et al 2008 |
| 104 | 3 months | alive at 3 months | Poor growth, hypotonia, hypertrichosis | CT: hypodensic areas in both cerebellar hemispheres and caudate nuclei | NA | NA | NA | NA | low | NA | c.841delCT | C.841delCT | Pronicki et al 2008 |
| 105 | 16 months | 10 years | Nystagmus, speech and walking difficulties | NA | NA | NA | NA | NA | low | NA | c.841delCT | NA | Pronicki et al 2008 |
| 106 | 9 months | alive at 9 months | Hypotonia, hypotonia, poor growth, vomiting, tremor | NA | NA | NA | NA | NA | NA | NA | c.841delCT | C.841delCT | Pronicki et al 2008 |
| 107 | 19 months | alive at 19 months | Tremor, ophthalmoplegia, dystonia, respiratory failure | CT: Symmetric hypodense areas of basal ganglia | NA | NA | NA | NA | low | NA | c.841delCT | C.841delCT | Pronicki et al 2008 |
| 108 | 12 months | alive at 12 months | Hypotonia, respiratory abnormalities, ophthalmoplegia, hypertrichosis | MRI at 2.5 years: symmetric hyperintense signals in basal ganglia | NA | NA | NA | NA | low | NA | c.841delCT | NA | Pronicki et al 2008 |
| 109 | 4 months | alive at 4 months | Motor regression, poor growth, hypotonia, tremor, hypertrichosis, ophthalmoplegia | NA | high | NA | NA | NA | low | NA | c.841delCT | C.841delCT | Pronicki et al 2008 |
| 110 | 2 months | alive at 2 months | Hypotonia, vomiting, respiratory abnormalities, ophthalmoplegia, ptosis, hypertrichosis | MRI at 4 years: symmetric hyperintense signals in basal ganglia, brain atrophy | NA | NA | NA | NA | low | NA | c.841delCT | C.841delCT | Pronicki et al 2008 |
| 111 | 7 months | alive at 7 months | Poor growth, vomiting, hypotonia, hypertrichosis, ophthalmoplegia | NA | NA | NA | NA | NA | low | NA | c.841delCT | C.841delCT | Pronicki et al 2008 |
| 112 | 14 months | alive at14 months | Motor regression, trembling, hypertrichosis, hypotonia | MRI: symmetric hyperintense signals in basal ganglia | NA | NA | NA | NA | low | NA | c.841delCT | C.841delCT | Pronicki et al 2008 |
| 113 | 7 months | alive at 7 months | Motor regression, respiratory abnormalities, vomiting | MRI: symmetric hyperintense signals in basal ganglia | high | NA | NA | NA | low | NA | c.841delCT | C.841delCT | Pronicki et al 2008 |
| 114 | 1st year | alive at 12 months | Poor growth, vomiting, tremor, respiratory abnormalities | NA | high | NA | NA | NA | low | NA | c.841delCT | NA | Pronicki et al 2008 |
| 115 | 6 months | alive at 6 months | Poor growth, hypotonia, respiratory abnormalities | MRI: symmetric hyperintense signals at lenticular nuclei, putamen, crura cerebri, substantia nigra, cerebellum periventricular areas,medulla oblongata | high | NA | NA | NA | low | NA | c.841delCT | C.841delCT | Pronicki et al 2008 |
| 116 | 2 years | alive at 8 years | Psychomotor development delay, hypotonia, ataxia, converging squint. Hypertrichosis | NA |  |  | Atrophic vacuolated fibres with numerous oil red O positive lipid droplets | low | NA | NA | NA | NA | Angelini et al 2009 |
| 117 | 16 months | 13 years 8 months | Hypotonia, speech and walking difficulties, nystagmus | NA | high | high | NA | NA | NA | NA | c.821A>G | c.845delCT | Piekutowska-Abramczuk et al 2009 |
| 118 | 20 months | 7 years 5 months | Progressive muscle atrophy, speech and walking disturbances, nystagmus | NA | NA | high | NA | NA | NA | NA | c.821A>G | c.845delCT | Piekutowska-Abramczuk et al 2009 |
| 119 | 36 months | alive at 60 month | Walking and speech difficulties, failure to thrive | NA | NA | NA | NA | NA | NA | NA | c.821A>G | c.845delCT | Piekutowska-Abramczuk et al 2009 |
| 120 | 12 months | 6 years and 7 month | Hypotonia, psychomotor retardation, muscle weakness | NA | NA | NA | NA | NA | NA | NA | c.821A>G, p.Tyr274Cys | c.845delCT | Piekutowska-Abramczuk et al 2009 |
| 121 | 24 months | 4 years and 5 months | Walking difficulties, hypotonia, nystagmus | NA | NA | NA | NA | NA | NA | NA | c.821A>G | c.845delCT | Piekutowska-Abramczuk et al 2009 |
| 122 | 24 months | alive 24 months | Walking difficulties, nystagmus | NA | NA | NA | NA | NA | NA | NA | c.821A>G | c.845delCT | Piekutowska-Abramczuk et al 2009 |
| 123 | 24 months | 20 years and 10 months | Walking difficulties, failure to thrive, echolalia | Lentiform nuclei right globus palladus, ventral medulla, enlarged ventricles, especially frontal horns | NA | NA | NA | NA | low | NA | c.704T>C | c.845delCT | Piekutowska-Abramczuk et al 2009 |
| 124 | 42 months | alive at 60 months | Hypotonia, mild psychomotor retardation | NA | NA | NA | NA | NA | low | low | c.574C>T | c.845delCT | Piekutowska-Abramczuk et al 2009 |
| 125 | 6 months | alive at 18 months | Jerky head movements, infantile spasm, developmental delay and regression | Moderate brain atrophy. No abnormal signal was detected in the white matter or in the nuclei | NA | NA | NA | NA | NA | NA | c.604G>C | c.604G>C | Xie et al 2009 |
| 126 | 6 months | alive at 1 year | Poor feeding, hypotonia, developmental regression | Moderate brain atrophy. No abnormal signal was detected in the white matter or in the nuclei | NA | NA | NA | NA | NA | NA | c.604G>C | c.604G>C | Xie et al 2009 |
| 127 | 3 years | 5 years | Hypotonia and ataxia | Bilateral putamen subthalamic nuclei substantia nigra, periaqueductal area, bilateral superior cerebellar peduncle, central tegmental tract, and dentate nuclei, the inferior olivary nuclei, dorsolateral medulla | NA | NA | NA | NA | NA | NA | c.604G>C | c.604G>C | Xie et al 2009 |
| 128 | 3 years | alive at 3 years | Ophthalmoplegia | Subthalamic nuclei, medial thalamic nuclei, midbrain, substantia nigra and central tegmental tract involved at superior colliculus level; tegmentum in pons and medulla | NA | NA | NA | NA | NA | NA | c.604G>C | c.604G>C | Xie et al 2009 |
| 129 | 3 years | alive at 12 years | Motor developmental delay, ophthalmoplegia, dysarthria | Moderate cerebral atrophy and severe cerebellar atrophy, hyperintense bilateral internal capsule and centrum semiovale | NA | NA | NA | NA | NA | NA | c.604G>C | c.604G>C | Xie et al 2009 |
| 130 | 1 year | alive at 8 years | Motor impairment and speech delay | Bilateral basal ganglia, subthalamic nuclei, and brain stem, centrum semiovale was abnormal | NA | NA | NA | NA | NA | NA | c.604G>C | c.604G>C | Xie et al 2009 |
| 131 | 1 year | alive at 12 years | Psychomotor delay, dysarthria, hypotonia, ataxia | NA | NA | NA | NA | NA | NA | NA | 604G>C | 604G>C | Xie et al 2009 |
| 132 | 1 year | alive at 8 years | Psychomotor delay, dysarthria, hypotonia, ataxia | NA | NA | NA | NA | NA | NA | NA | 604G>C | 604G>C | Xie et al 2009 |
| 133 | 14 months | 22 months | Developmental delay, hypotonia, poor growth, neurodevelopmental regression | Substantia nigra and subthalamic nucleus | high | NA | High lipid | low | low | low | c.240 +1G > T | C534delAAAT | Bruno et al 2009 |
| 134 | 13 months |  | Partial seizures, ataxia, nystagmus, right optic atrophy, hypotonia | Cerebellar hypodensities in white matter and dentate nuclei | high | NA | NA | NA | NA | NA | NA | NA | Timothy et al 2009 |
| 135 | NA | NA | Poor growth, ataxia, hypertrichosis | Basal ganglia | high | NA | NA | NA | NA | NA | c.312_321del10insAT | c.312_321del10insAT | Lee et al 2012 |
| 136 | NA | NA | Poor growth, myopathy, hypertrichosis | Basal ganglia | high | NA | NA | NA | NA | NA | c.312_321del10insAT | c.312_321del10insAT | Lee et al 2012 |
| 137 | NA | NA | Poor growth, seizure | Basal ganglia | high | NA | NA | NA | NA | NA | c.240+1G>T | c.516-2A>G | Lee et al 2012 |
| 138 | NA | NA | Poor growth | Basal ganglia | high | NA | NA | NA | NA | NA | c.312_321del10insAT (p.L105X) | c.312_321del10insAT (p.L105X) | Lee et al 2012 |
| 139 | NA | NA | Nystagmus | Subthalamic nuclei, medulla, tegmentum | high | NA | NA | low | low | NA | c.845_846delCT | c.269T>C | Lee et al 2012 |
| 140 | NA | NA | Short stature, poor growth, ataxia, ophthalmoplegia, sensorineural hearing loss, peripheral neuropathy | Basal ganglia | normal | NA | NA | NA | NA | NA | c.653_654delCT | c.807_810del4ins9 | Lee et al 2012 |
| 141 | NA | NA | Seizures, ptosis, muscle weakness, hypotonia, dysmorphism | Leukodystrophy | high | NA | NA | NA | NA | NA | c.169delG | c.530T>G | Lee et al 2012 |
| 142 | NA | NA | Poor growth, hypotonia, microcephaly | Leukodystrophy | normal | NA | NA | NA | NA | low | c.324-11T>G | c.324-11T>G | Lee et al 2012 |
| 143 | NA | Alive at 3.5 years | Spastic diparesis, swallowing difficulty, ataxia, seizure, hypertrichosis | Basal ganglia | high | NA | NA | NA | NA | low | c.555_556delGA | c.769G>A | Lee et al 2012 |
| 144 | NA | NA | Poor growth | Leukodystrophy | high | NA | NA | NA | NA | low | c.555_556delGA | c.574_575insCTGC | Lee et al 2012 |
| 145 | NA | NA | NA | NA | NA | NA | NA | NA | NA | low | c.312_321del10insAT | c.574_575insCTGC | Lee et al 2012 |
| 146 | NA | Died at 4.5 years | Hypotonia, ataxia, ophthalmoplegia, nystagmus | Basal ganglia, cerebellar and medulla | high | NA | NA | NA | NA | NA | c.574_575insCTGC | c.55-1G>A | Lee et al 2012 |
| 147 | NA | Alive at 6 years | Poor growth, hypotonia, muscle weakness, ataxia, swallowing difficulties, VI nerve palsy, hypertrichosis | Basal ganglia | high | NA | NA | NA | NA | NA | c.312_321del10insAT | c.614G>A | Lee et al 2012 |
| 148 | NA | Alive at 4 years | Poor growth, hypotonia, muscle weakness, swallowing difficulties, cardiovascular malformations, Down syndrome | Basal ganglia, midbrain | high | NA | NA | NA | NA | NA | c.312_321del10insAT | c.614G>A | Lee et al 2012 |
| 149 | NA | NA | NA | NA | NA | NA | NA | NA | NA | NA | c.833+1G>A | c.833+1G>A | Lee et al 2012 |
| 150 | NA | Alive at 4 years | Poor growth, hypotonia, ataxia, apnoea | Basal ganglia | high | NA | NA | NA | NA | NA | c.183_186delTCTT | c.312_321del10insAT | Lee et al 2012 |
| 151 | NA | Died at 3.5 years | Poor growth, hypotonia, ataxia, myoclonic jerks, extrapyramidal movements | Basal ganglia, periaqueductal grey matter | high | NA | NA | NA | NA | NA | c.106+1G>C | c.106+1G>C | Lee et al 2012 |
| 152 | NA | Alive at 3 years | Poor growth, hypotonia, ataxia, dystonia | Cerebellum, brainstem, and basal ganglia | high | NA | NA | NA | NA | NA | c.845_846delCT | c.107-2A>G | Lee et al 2012 |
| 153 | NA | Alive at 5 years | Hypotonia, ataxia, nystagmus | Brainstem, cerebellum, basal ganglia, and right occipital white matter lesions | high | NA | NA | NA | NA | NA | c.472_473delAG | c.845delCT | Lee et al 2012 |
| 154 | NA | Alive, at 2 years | Poor growth, hypotonia, muscle weakness | Basal ganglia, leukodystrophy | high | NA | NA | NA | NA | NA | c.312_321del10insAT (p.L105X) | c.845_846delCT (p.S282CfsX9) | Lee et al 2012 |
| 155 | NA | Alive, at 3 years | Poor growth, hypotonia, chorea, cortical visual impairment, microcephaly, myoclonic epilepsy, gastro-oesphageal reflux, constipation, elevatedtransaminases, cataract, hypogammaglobulinaemia | Generalized volume loss and hypomyelination | NA | NA | NA | NA | low | NA | c.167C>G (p.A56G) | c.751+6T>C | Lee et al 2012 |
| 156 | NA | NA | Developmental arrest, facial dysmorphism, hypertrichosis, Mild opthalmoplegia  ptosis, mild hypotonia, truncal ataxia | Hyperintensity of the bilateral putamen, subthalamic nucleus, red nucleus and brain stem | high | NA | NA | NA | NA | NA | c.49+1 G>T | c.752_753del | Tanigawa 2012 |
| 157 | 19 months | alive at 8 years |  | Bilateral cerebral hemispheres globally atrophic, hyperintensity of bilateral optic radiation, putamen, basal ganglia including subthalamic nucleus, and globus pallidus. | NA | high | NA | NA | NA | low | c.743 C>A, p.Ala248Asp | c.743 C>A, p.Ala248Asp | Tanigawa 2012 |

*Mutation data is reported using the nomenclature used in the original papers; NA data not available

**References**

Bruno C, Biancheri R, Garavaglia B, Biedi C, Rossi A, Lamba LD, et al. A novel mutation in the SURF1 gene in a child with Leigh disease, peripheral neuropathy, and cytochrome-c oxidase deficiency. J Child Neurol 2002, 17:233-6

Böhm M, Pronicka E, Karczmarewicz E, Pronicki M, Piekutowska-Abramczuk D, Sykut-Cegielska J, et al. Pediatr Res 2006, 59:21-6

Coenen MJ, Smeitink JA, Pots JM, van Kaauwen E, Trijbels FJ, Hol FA,et al. Sequence analysis of the structural nuclear encoded subunits and assembly genes of cytochrome c oxidase in a cohort of 10 isolated complex IV-deficient patients revealed five mutations. J Child Neurol 2006, 21:508-11

Coenen MJ, Smeitink JA, Farhoud MH, Nijtmans LG, Rodenburg R, Janssen A, et al. The first patient diagnosed with cytochrome c oxidase deficient Leigh syndrome: progress report. J Inherit Metab Dis 2006, 29:212-3

Darin N, Moslemi AR, Lebon S, Rustin P, Holme E, Oldfors A, et al. Genotypes and clinical phenotypes in children with cytochrome-c oxidasedeficiency. Neuropediatrics 2003, 34:311-7. Review.

Head RA, Brown RM, Brown GK. Diagnostic difficulties with common SURF1 mutations in patients with cytochrome oxidase-deficient Leigh syndrome. J Inherit Metab Dis 2004, 27:57-65.

Lee IC, El-Hattab AW, Wang J, Li FY, Weng SW, Craigen WJ, Wong LJ. SURF1-associated Leigh syndrome: a case series and novel mutations. Hum Mutat 2012, 33:1192-1200

Moslemi AR, Tulinius M, Darin N, Aman P, Holme E, Oldfors A. SURF1 gene mutations in three cases with Leigh syndrome and cytochrome c oxidase deficiency. Neurology 2003, 61:991-3

Ostergaard E, Bradinova I, Ravn SH, Hansen FJ, Simeonov E, Christensen E, et al. Hypertrichosis in patients with SURF1 mutations. Am J Med Genet A 2005, 138:384-8.

Pecina P, Houstková H, Hansíková H, Zeman J, Houstek J. Genetic defects of cytochrome c oxidase assembly. Physiol Res 2004, 53 Suppl 1:S213-23. Review.

Pecina P, Capková M, Chowdhury SK, Drahota Z, Dubot A, Vojtísková A, Hansíková H, Houst'ková H, Zeman J, Godinot C, Houstek J. Functional alteration of cytochrome c oxidase by SURF1 mutations in Leigh syndrome. Biochim Biophys Acta 2003,1639:53-63.

Pequignot MO, Desguerre I, Dey R, Tartari M, Zeviani M, Agostino A et al. New splicing-site mutations in the SURF1 gene in Leigh syndrome patients. J Biol Chem 2001, 276:15326-9

Piekutowska-Abramczuk D, Popowska E, Pronicki M, Karczmarewicz E, Tylek-Lemanska D, Sykut-Cegielska et al. High prevalence of SURF1 c.845_846delCT mutation in Polish Leigh patients. Eur J Paediatr Neurol 2009, 13:146-53

Piekutowska-Abramczuk D, Magner M, Popowska E, Pronicki M, Karczmarewicz E, Sykut-Cegielska J,et al. SURF1 missense mutations promote a mild Leigh phenotype. Clin Genet 2009, 76:195-204

Piekutowska-Abramczuk D, Popowska E, Pronicka E, Karczmarewicz E, Pronicki M, Kmieć T, et al. SURF1 gene mutations in Polish patients with COX-deficient Leigh syndrome. J Appl Genet 2001, 42:103-8.

Poyau A, Buchet K, Bouzidi MF, Zabot MT, Echenne B, Yao J, et al. Missense mutations in SURF1 associated with deficient cytochrome c oxidase assembly in Leigh syndrome patients. Hum Genet 2000, 106:194-205.

Poyau A, Buchet K, Godinot C. Sequence conservation from human to prokaryotes of Surf1, a protein involved in cytochrome c oxidase assembly, deficient in Leigh syndrome. FEBS Lett 1999, 462:416-20.

Pronicki M, Matyja E, Piekutowska-Abramczuk D, Szymanska-Debinska T, Karkucinska-Wieckowska A, Karczmarewicz E, et al. Light and electron microscopy characteristics of the muscle of patients with SURF1 gene mutations associated with Leigh disease. J Clin Pathol 2008, 61:460-6

Péquignot MO, Dey R, Zeviani M, Tiranti V, Godinot C, Poyau A, et al. Mutations in the SURF1 gene associated with Leigh syndrome and cytochrome C oxidase deficiency. Hum Mutat 2001,17:374-81

Rahman S, Brown RM, Chong WK, Wilson CJ, Brown GK. A SURF1 gene mutation presenting as isolated leukodystrophy. Ann Neurol 2001, 49:797-800

Rossi A, Biancheri R, Bruno C, Di Rocco M, Calvi A, Pessagno A, et al. Leigh Syndrome with COX deficiency and SURF1 gene mutations: MR imaging findings. AJNR Am J Neuroradiol 2003, 24:1188-91

Sacconi S, Salviati L, Trevisson E. Mutation analysis of COX18 in 29 patients with isolated cytochrome c oxidase deficiency. J Hum Genet 2009, 54:419-21

Sacconi S, Salviati L, Sue CM, Shanske S, Davidson MM, Bonilla E, et al. Mutation screening in patients with isolated cytochrome c oxidase deficiency. Pediatr Res 2003, 53:224-30

Salviati L, Freehauf C, Sacconi S, DiMauro S, Thoma J, Tsai AC. Novel SURF1 mutation in a child with subacute encephalopathy and without the radiological features of Leigh Syndrome. Am J Med Genet A 2004, 128A:195-8

Santoro L, Carrozzo R, Malandrini A, Piemonte F, Patrono C, Villanova M, et al. A novel SURF1 mutation results in Leigh syndrome with peripheral neuropathy caused by cytochrome c oxidase deficiency. Neuromuscul Disord 2000, 10:450-3

Savasta S, Comi GP, Perini MP, Lupi A, Strazzer S, Rognoni F, Rossoni R. Leigh disease: clinical, neuroradiologic, and biochemical study of three new cases with cytochrome c oxidase deficiency. J Child Neurol. 2001, 16:608-13.

Savoiardo M, Zeviani M, Uziel G, Farina L. MRI in Leigh syndrome with SURF1 gene mutation. Ann Neurol 2002, 51:138-9

Tanigawa J, Kaneko K, Honda M, Harashima H, Murayama K, Wada T, et al. Two Japanese patients with Leigh syndrome caused by novel SURF1 mutations. Brain Dev 2012, 34:861-5

Tay SK, Sacconi S, Akman HO, Morales JF, Morales A, De Vivo DC, et al. Unusual clinical presentations in four cases of Leigh disease, cytochrome C oxidase deficiency, and SURF1 gene mutations. J Child Neurol 2005, 20:670-4

Teraoka M, Yokoyama Y, Ninomiya S, Inoue C, Yamashita S, Seino Y. Two novel mutations of SURF1 in Leigh syndrome with cytochrome c oxidase deficiency. Hum Genet 1999, 105:560-3

Timothy J, Geller T. SURF-1 gene mutation associated with leukoencephalopathy in a 2-year-old. J Child Neurol 2009,24:1296-301

Tiranti V, Hoertnagel K, Carrozzo R, Galimberti C, Munaro M, Granatiero M, et al. Mutations of SURF-1 in Leigh disease associated with cytochrome c oxidase deficiency. Am J Hum Genet 1998, 63:1609-21

Tiranti V, Jaksch M, Hofmann S, Galimberti C, Hoertnagel K, Lulli L, et al. Loss-of-function mutations of SURF-1 are specifically associated with Leigh syndrome with cytochrome c oxidase deficiency. Ann Neurol 1999, 46:161-6.

van Riesen AK, Antonicka H, Ohlenbusch A, Shoubridge EA, Wilichowski EK. Maternal segmental disomy in Leigh syndrome with cytochrome c oxidase deficiency caused by homozygous SURF1 mutation. Neuropediatrics 2006, 37:88-94

Von Kleist-Retzow JC, Yao J, Taanman JW, Chantrel K, Chretien D, Cormier-Daire V, et al. Mutations in SURF1 are not specifically associated with Leigh syndrome. J Med Genet 2001, 38:109-13

von Kleist-Retzow JC, Vial E, Chantrel-Groussard K, Rötig A, Munnich A, Rustin P, et al. Biochemical, genetic and immunoblot analyses of 17 patients with an isolated cytochrome c oxidase deficiency. Biochim Biophys Acta 1999, 1455:35-44

Williams SL, Valnot I, Rustin P, Taanman JW. Cytochrome c oxidase subassemblies in fibroblast cultures from patients carrying mutations in COX10, SCO1, or SURF1. J Biol Chem 2004, 279:7462-9

Williams SL, Taanman JW, Hansíková H, Houst'ková H, Chowdhury S, Zeman J, et al. A novel mutation in SURF1 causes skipping of exon 8 in a patient with cytochrome c oxidase-deficient leigh syndrome and hypertrichosis. Mol Genet Metab 2001, 73:340-3

Xie S, Xiao JX, Qi ZY, Yang YL, Jiang XX. Heterogeneity of magnetic resonance imaging in Leigh syndrome with SURF1 gene 604G-->C mutation. Clin Imaging 2009, 33:1-6

Yang YL, Sun F, Zhang Y, Qian N, Yuan Y, Wang ZX, et al. Clinical and laboratory survey of 65 Chinese patients with Leigh syndrome. Chin Med J (Engl). 2006, 119:373-7

Yüksel A, Seven M, Cetincelik U, Yeşil G, Köksal V. Facial dysmorphism in Leigh syndrome with SURF-1 mutation and COX deficiency. Pediatr Neurol 2006, 34:486-9

Zhang Y, Yang YL, Sun F, Cai X, Qian N, Yuan Y, et al. Clinical and molecular survey in 124 Chinese patients with Leigh or Leigh-like syndrome. J Inherit Metab Dis 2007, 30:265

Zhu Z, Yao J, Johns T, Fu K, De Bie I, Macmillan C, et al. SURF1, encoding a factor involved in the biogenesis of cytochrome c oxidase, is mutated in Leigh syndrome. Nat Genet 1998, 20:337-43
